# Hempseed Lignanamides Rich-Fraction: Chemical Investigation and Cytotoxicity towards U-87 Glioblastoma Cells

**DOI:** 10.3390/molecules25051049

**Published:** 2020-02-26

**Authors:** Ersilia Nigro, Giuseppina Crescente, Marialuisa Formato, Maria Tommasina Pecoraro, Marta Mallardo, Simona Piccolella, Aurora Daniele, Severina Pacifico

**Affiliations:** 1 Department of Environmental Biological and Pharmaceutical Sciences and Technologies, University of Campania “Luigi Vanvitelli”, Via Vivaldi 43, I-81100 Caserta, Italy; nigro@ceinge.unina.it (E.N.); giuseppina.crescente@unicampania.it (G.C.); marialuisa.formato@unicampania.it (M.F.); mariatommasina.pecoraro@unicampania.it (M.T.P.); marta.mallardo@unicampania.it (M.M.); simona.piccolella@unicampania.it (S.P.); aurora.daniele@unicampania.it (A.D.); 2 CEINGE-Advanced Biotechnologies, Scarl, 80131 Napoli, Italy

**Keywords:** *Cannabis sativa* L., phenylamides, lignanamides, hemp seeds, high resolution tandem mass spectrometry, U-87 glioblastoma cells, cytotoxicity

## Abstract

The weak but noteworthy presence of (poly)phenols in hemp seeds has been long overshadowed by the essential polyunsaturated fatty acids and digestible proteins, considered responsible for their high nutritional benefits. Instead, lignanamides and their biosynthetic precursors, phenylamides, seem to display interesting and diverse biological activities only partially clarified in the last decades. Herein, negative mode HR-MS/MS techniques were applied to the chemical investigation of a (poly)phenol-rich fraction, obtained from hemp seeds after extraction/fractionation steps. This extract contained phenylpropanoid amides and their random oxidative coupling derivatives, lignanamides, which were the most abundant compounds and showed a high chemical diversity, deeply unraveled through high resolution tandem mass spectrometry (HR-MS/MS) tools. The effect of different doses of the lignanamides-rich extract (LnHS) on U-87 glioblastoma cell line and non-tumorigenic human fibroblasts was evaluated. Thus, cell proliferation, genomic DNA damage, colony forming and wound repair capabilities were assessed, as well as LnHS outcome on the expression levels of pro-inflammatory cytokines. LnHS significantly inhibited U-87 cancer cell proliferation, but not that of fibroblasts, and was able to reduce U-87 cell migration, inducing further DNA damage. No modification in cytokines’ expression level was found. Data acquired suggested that LnHS acted in U-87 cells by inducing the apoptosis machinery and suppressing the autophagic cell death.

## 1. Introduction

Plant foods, thanks to the functionality of their bioactive secondary metabolites, are considered to be both safe and able to promote good health [1], explaining some important targeted effects in humans, and preventing affluence diseases, such as cardiovascular diseases and cancers [2]. Bioactive compounds are also in ancient crops, whose actual recovery further renewed the phytochemical research into the discovery of beneficial substances, which could be present, after food processing, in the daily meals or be exceptionally lost in produced waste and by-products [3]. This is the case of lignans, chemically characterized by a phenylpropanoid core, which are reported to exert numerous biological effects in mammals, including antitumor and antioxidant activities [4]. They act as phytoestrogens and are converted by intestinal microflora into mammalian lignans or enterolignan compounds [5].

Lignans are abundant in dietary sources like whole-grain cereals and legumes. The preventive benefits of some edible seeds, and the increasing intake of no-conventional foods as chia, quinoa, flax, canola and pumpkin seeds could be ascribed to their richness in these compounds [6]. For instance, flaxseed (*Linum usitatissimum* L.) is reported to contain about 75–800 times more lignans than cereal grains, legumes, fruits and vegetables, together with antioxidant flavonols and hydroxycinnamic acids [7], and lariciresinol was one of the main constituents of pumpkin seeds (*Cucurbita pepo* L.) [8].

Hemp seeds (non-drug type of *Cannabis sativa* L.) also contain, beyond proteins, and polyunsaturated fatty acids, bioactive lignan derivatives, known as lignanamides [9,10,11,12]. These latter could be found in hemp fruits together with their biosynthetic precursors, namely phenylamides, whose presence is functional in another seed, such as oat seed, which produces avenanthramides (AVAs) with important anti-inflammatory and antiproliferative effects [13]. Until a few years ago, phenylamides and lignanamides seemed to constitute a small group of natural products, whose distribution in plant kingdom was thought to be limited to plants of the Cannabaceae and Solanaceae family. Indeed, lignanamides are also reported from *Mitrephora thorelii* (Annonaceae) and *Corydalis saxicola* (Papaveraceae), and, recently, five pairs of enantiomeric lignanamides were obtained from *Solanum nigrum*, and melongenamides A–D were isolated from the roots of *Solanum melongena* L. [14,15,16]. Furthermore, new lignanamides and neolignanamides were isolated from *Lycium chinense* [17,18], highlighting that the diversity of these compounds is so far to be really known. Moreover, (±)-sativamides A and B, two pairs of nor-lignanamide enantiomers featuring a unique benzo-angular triquinane skeleton, were isolated from the fruits of *Cannabis sativa*, and were observed to be able to reduce endoplasmic reticulum (ER) stress-induced cytotoxicity in neuroblastoma cells [19].

The ability of lignanamides to display interesting and diverse biological activities, including feeding deterrent activity, insecticidal effects, anti-inflammatory and neuroprotective activity [20,21], addresses the research in new analytical challenges for their ready exploitation from hempseed meal. In this context, starting on the great and renewed interest in hemp seeds and their by-products, as source of essential nutrients, in sufficient amount and ratio to satisfy the dietary human demand, commercial hempseeds underwent ultrasound assisted extraction first with *n*-hexane, and after an oil-like mixture recovery, the obtained by-product was investigated for its polyphenol content. A fraction rich in lignanamides (LnHS) was achieved and chemically profiled through HR-MS/MS tools operating in negative ion detection. Furthermore, taking into account recent literature, which highlights the protective effects of pure lignanamides on central nervous system cell lines [15,21], LnHS was investigated for its anti-cancer properties versus U-87 malignant glioblastoma (GMB) neuroepithelial cells. Indeed, U-87 cells are known to move very fast and to aggregate as clusters, showing rapid migration, and highest invasion ability [22]. As malignant gliomas are the most common primary brain tumors, among which GBM is the most malignant and highly aggressive [23], the aim of this study was to understand the effects of low doses of constituted LnHS fraction on U-87 viability cell line as well-established model of malignant glioblastoma cell line in terms of apoptosis and autophagic cell death. In addition, the effects of LnHS on U-87 colony formation efficacy and cell migration was analyzed. Finally, the ability of LnHS of inducing oxidative and inflammation processes was investigated through evaluation of the expression of Sirt1 and Sirt2, as well as of some pro-inflammatory cytokines. All tests were performed in comparison to non-tumorigenic human fibroblasts (thereafter indicated as HF).

## 2. Results and Discussion

The interest in the polyphenols contained in hemp seed was for a long time obscured by its high content in essential polyunsaturated fatty acids, mainly linoleic and α-linolenic acids, though different flavonoid glycosides were identified in cold-pressing hemp seed oil [24,25]. Indeed, after oil recovery, hemp seed meal yet represents an important food polyphenol source. Several phenylamides and lignanamides were previously isolated and structurally identified mainly by means of nuclear magnetic resonance (NMR) spectroscopic tools [20,26]. Herein, in order to get insight into the chemical composition of commercial hempseeds, ultrasound assisted maceration was applied on seeds first ground with a knife mill and crushed into liquid N_2_ to better preserve the integrity of the fruit’s constituents.

The cryo-crushed matrix, made as a friable powder, preliminarily underwent solid-liquid extraction process using *n*-hexane, obtaining an oil-like extract and the defatted-matrix. This latter was then ultrasound-assisted macerated using ethanol (Figure S1). The alcoholic fraction was further fractionated (please see Section 3) obtaining, among the others, a fraction, hereafter referred to as LnHS, able to strongly modify the morphology of SH-SY5Y cells (Figure S2). Thus, in the consciousness that extraction/fractionation steps are fundamental into defining the chemistry of a phytochemical extract, in order to get insight into this fraction chemical composition, negative HR-MS and HR-MS/MS spectra, as well as spectra by ultraviolet diode array detection (UV-DAD), were acquired. Phenylamides and lignanamides were found to be the main compounds (Table 1 and Table 2), whereas flavonol glycosides were the minor constituents (Table 3). Thus, the fraction underwent an extensive cytotoxic screening on U-87 cells, exhibiting a promising behavior in fighting migration and invasion features of these glioblastoma cells.

### 2.1. HR-MS Analysis: Phenylamides

**Figure 1:**
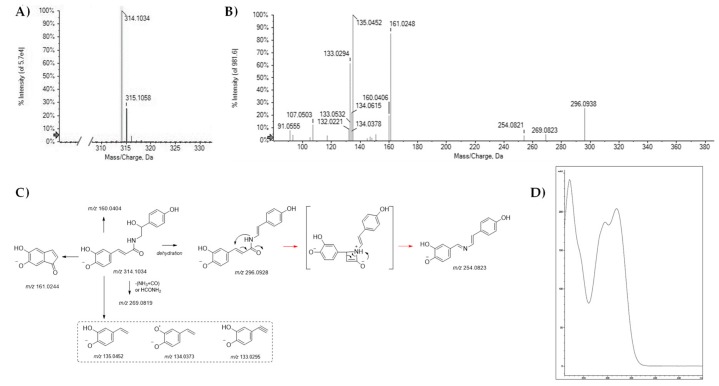
(**A**) and (**B**) TOF-MS and TOF-MS/MS spectra for compound **1**; (**C**) proposed fragmentation pathway of the [M − H]^−^ ion; (**D**) UV-DAD spectrum. In **C** panel, the theoretical *m/z* value is reported below each structure.

Compounds **1**, **2**, **5**, **8**, **11**, **17** and **33** were recognized as phenylamides, conjugates of aliphatic polyamines or arylmonoamines and hydroxycinnamic acids, suggested as defensive plant-specific molecules. Compound **1** was tentatively identified as *N*-caffeoyloctopamine (Figure 1), previously isolated from hempseed cakes in a screening aimed to identify novel arginase inhibitors [27], and further identified among hempseed constituents with potential anti-neuroinflammatory activity [25].

The [M − H]^−^ ion at *m/z* 314.1039 underwent dehydration (likely through octopamine moiety) providing the TOF-MS^2^ fragment ion at *m/z* 296.0910, or rearranged to give, through 45 Da neutral loss, the fragment ion at *m/z* 269.0818.

This could be due to (NH_3_ + CO) neutral loss eliminated in one step (as HCONH_2_), or, more likely, eliminated in rapid sequential steps [28]. The N-CO α-cleavage, a characteristic fragmentation observed as a common pattern in all natural and synthetic amides [29], could drive the genesis of the ion at *m/z* 161.0244, whereas ions at *m/z* 135.0455 (base peak), 134.0373, and 133.0302, and 132.0217 were attributable to dihydroxycinnamoyl residue (Table 1). UV-DAD spectra of compound **1** showed absorption bands at 324, 296, and 216 nm, plus a shoulder at 242 nm. This latter, together with the broad band at 324 nm was attributable to aromatic moieties π→π* transitions, whereas the n→π* and π→π* electronic transitions referred to the amidic group were responsible for the absorption at 296 and 216 nm. Compound **2** showed the [M − H]^−^ ion at *m/z* 298.1086 according to the molecular formula C_17_H_17_NO_4_. TOF-MS^2^ spectrum suggested the occurrence of *N*-*p*-coumaroyloctopamine, before reported as an inducible phenolic amide in potato tuber tissue [30]. The deprotonated molecular ion gave rise to the ion [M − H − H_2_O]^−^ at *m/z* 280.0981, which in turn provided the ions at *m/z* 134.0607 and 119.0502. The ion at *m/z* 145.0293 confirmed coumaroyl moiety, as well as the fragment ion at *m/z* 160.0408, which could be from the cleavage at the N-Cα bond and the following 1,4 nucleophilic addition on the β’-carbon of the phenylpropanoid side chain. The presence of the coumaroyl moiety was further revealed through the UV-DAD spectrum of the compound, which is was similar to that previously reported for this hydroxycinnamoyl amide (HAA) [26] and it was consistent with the loss of catechol group as highlighted by the blue shift of the absorption band detectable at 324 nm in compound **1** (Figure S3).

**Figure 2:**
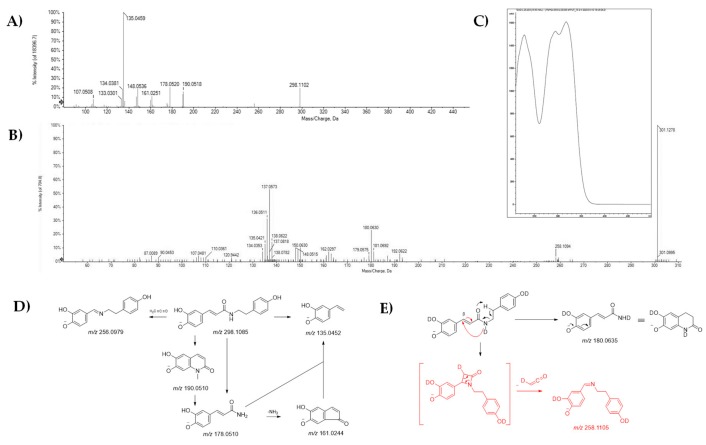
TOF-MS/MS spectra of compound 5 (**A**) and of its 3d-derived (**B**). UV-DAD spectrum of the compound (**C**). Proposed fragmentation pathway of the [M − H]^−^ ion for compound **5** (**D**) and the 3*d*-derived (**E**); theoretical *m/z* value is reported below each structure.

A constitutional isomer of the previous compound was *N*-caffeoyltyramine, which was found to inhibit macrophage-mediated inflammatory responses through the suppression of the production of NO and pro-inflammatory cytokines [31]. This compound was tentatively identified under peak **5**. In this case, the [M − H]^−^ ion at *m/z* 298.1090 dissociated providing the TOF-MS^2^ fragment ion at *m/z* 135.0459 as base peak, likely corresponding to 2-hydroxy-4-vinylphenolate. Following the cleavage at the N-Cα bond, the fragment ion at *m/z* 178.0520 was formed, whereas the ion at *m/z* 190.0518 could be from Cα-Cβ bond breakdown [32]. The CH_2_CO loss likely consisted in the fragment ion at *m/z* 256.0974 (Figure 2). In order to corroborate this latter hypothesis, hydrogen–deuterium (H/D) exchange reaction was carried out on pure *N*-caffeoyltyramine. The TOF-MS^2^ spectra of the 3*d*-derived underwent rearrangement to give the ion at *m/z* 180.0619, whereas the loss of an ethen-1-one-2-d provided the fragment ion at *m/z* 258.1098. The electronic absorption spectrum of compound **5** (Figure 2C), similarly to that of compound **1**, with which shared the common caffeoyl component, evidenced the bathochromic and hyperchromic effect of -OH functional auxochromic groups of the double absorption band, which was at 317 and 294 nm, whereas the other band was at 237 nm. The compound was a constituent of other inestimable food sources such as hot pepper (*Capsicum annuum*), a spice used worldwide [33], Goji berry, the fruit of *Lycium barbarum* [34] and seeds of *Annona crassiflora* Mart., a fruitful tree native to the Brazilian Cerrado biome [35].

Compounds **8** and **17**, which showed the [M − H]^−^ ion at *m/z* 312.1243 and 312.1241, respectively, were tentatively identified as *N*-feruloyltyramine geometrical isomers (Figure S4). The deprotonated molecular ion underwent in both TOF-MS^2^ spectra methyl radical loss to achieve the fragment ion at *m/z* 297.1015(4), which gave rise to fragment ions at *m/z* 190.0510(3) and 178.0513, or more favorably led to ion at *m/z* 148.0530(5) (base peak) through CO-Cα’ bond cleavage. The CH_3_^●^ loss generated the radical ion at *m/z* 134.0373(6), as well as the ion at *m/z* 135.0452(6).

The [M − H]^−^ ion at *m/z* 282.1140 for compound **11** was in accordance with the molecular formula C_17_H_17_NO_4_. UV-DAD and TOF-MS/MS spectra were according to *N*-*p*-coumaroyltyramine, an antioxidant HAA compound with inhibiting effect on acetylcholinesterase, cell proliferation, platelet aggregation [36]. In particular, in TOF-MS/MS spectrum, beyond the fragment ion at *m/z* 162.0558, which was consistent with coumaramide, the abundance of 4-vinylphenolate further confirmed the acylic moiety identity (Figure S5). Finally, compound **33** was tentatively identified as tri-*p*-coumaroylspermidine (Figure S6). This latter, until now never reported among hemp seed constituents, was firstly reported to reduce the mycelial growth of the oat leaf stripe pathogen *Pyrenophora avenae* and also the infective ability of powdery mildew (*Blumeria graminis* f. sp. hordei) [37], and recently described as constituent of *Salvia* and *Lavandula* species [38].

### 2.2. HR-MS Analysis: Lignanamides

The great part of the other identified compounds belongs to the lignanamide class (Table 2), which in *Cannabis sativa* fruit appears highly variable. In fact, it was reported to be constituted by aryl(dihydro)naphthalene-type, benzodioxanes-type and β-arylether-type compounds, as well as nor-lignanamides with a peculiar benzo-angular triquinane skeleton [10]. The arylnaphthalene lignanamide, cannabisin A, and three phenyldihydronaphthalene lignanamides were among the first isolated compounds in hemp fruits, and were herein tentatively identified thanks to their abundance, which made them easily isolable, and common fragmentation pattern features. Furthermore, UV-DAD spectra were also acquired and compared to those of pure standard compounds. In particular, cannabisin A, an arylnaphthalene lignanamide isolated so far from fruits of *Cannabis sativa* [39], was putatively identified in compound **16** (Figure S7). The [M − H]^−^ ion at *m/z* 593.1941 (error ppm 2.0), in accordance with the molecular formula C_34_H_30_N_2_O_8_, gave the fragment ion at *m/z* 456.1110, which in turn, thanks to CO neutral loss, provided the ion at *m/z* 428.1151. The loss of 163.06 Da, likely corresponding to isocyanic acid (HNCO) + *p*-hydroxystyrene, also could directly occur from the deprotonated molecular ion supplying the fragment ion at *m/z* 430.1313. As previously reported, it appeared as HNCO elimination competes with backbone cleavage [40]. To support the hypothesis of *p*-hydroxystyrene residue loss, the ion at *m/z* 336.0520 was observed to be formed, which in turn, after HNCO loss, gave the ion at *m/z* 293.0457. This latter could be also obtained from the base peak due to 163.06 Da loss. UV-DAD spectrum of peak **16** was fully comparable to that previously reported for the pure reference compound [26]. In fact, UV-DAD spectrum exhibited a characteristic strong maximum at 256 nm and only small peaks between 280 and 350 nm.

**Figure 3:**
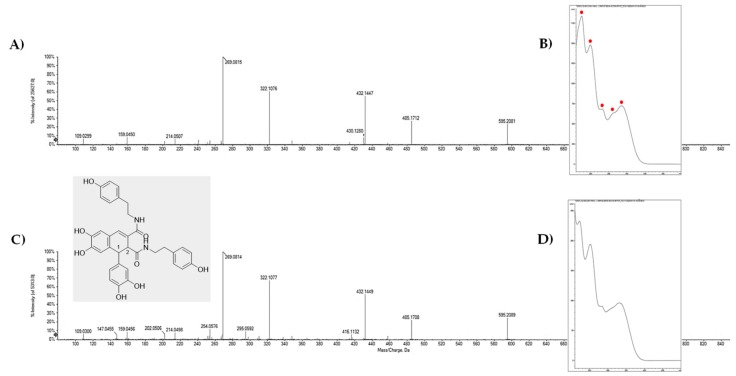
TOF-MS/MS spectra of compounds **15** and **22** (**A** and **C**, respectively). UV-DAD spectra of the compounds (**B** and **D**). In grey panel, the structure of cannabisin B is reported, without emphasizing stereochemical features.

The [M − H]^−^ ion of compounds under peaks **15**, **22**, **28**, **32**, **36** and **38** was in line with the C_34_H_32_N_2_O_8_ molecular formula and calculated exact mass equal to *m/z* 595.2086. TOF-MS/MS spectra of **15** and **22** were almost super-imposable, and their UV-DAD spectra mostly resembled the previously reported for cannabisin B electronic absorptions [26], with maximums at 218, 254, 282, 310 and 342 nm (Figure 3 and Figure S8). In particular, TOF-MS/MS spectra showed the ions at *m/z* 269.08 as base peak, whereas the ions at *m/z* 485.17 (− catechol), 432.14 (likely due to the loss of HNCO + *p*-hydroxystyrene), and 322.10, which could be favorably formed when the moiety weighing 163.06 Da was lost together with a catechol unit, were detected with their relative high abundance. Cannabisin B, among lignanamides from hempseed, was one of the first to be investigated for its biological behavior, and it was found that it showed a marked antiproliferative action on HepG2 cell [11].

Compound **28** was likely 3,3′-didemethyl-grossamide. The [M − H]^−^ ion dissociated, as observed in the TOF-MS/MS experiment, providing the ion *m/z* 458.1262, attributable to a tyramine moiety direct neutral loss, as well as the more abundant ion at *m/z* 432.1468. This latter could further loss a tyramine unit providing the ion at *m/z* 295.0616 or also, more favorably supplied the base peak at *m/z* 269.0828, which was in accordance with a 2-hydroxy-4-(7-hydroxy-5-vinyl-2,3-dihydrobenzofuran-2-yl)phenolate (Figure S9). The occurrence of a phenylcoumaran lignandiamide with 2,3-dihydrobenzofuran nucleus was supposed, and it could be derived through the 8-5′ coupling of two caffeoyl alcohols [41]. The comparison of the UV-DAD spectrum with that reported in literature allowed us to putatively further identify the compound [26]. Finally, the [M − H]^−^ ion of compound under peak **32**, which fragmented into the base peak at *m/z* 298.1083, and minor fragment ions at *m/z* 178.0503 and 135.0444, could be attributable to 3,3′-demethyl-heliotropamide, an oxopyrrolidine-3-carboxamide, previously isolated from hemp fruits [20], whereas the benzodioxanes-type lignandiamides, cannabisin M and cannabisin Q, were tentatively identified from TOF-MS/MS spectra of compounds **36** and **38** (Figure S10). Charge-driven collision-induced dissociation could favor the formation of a phenoxide anion, which retro-cleaved leading the fragment ion at *m/z* 298.1088 (base peak).

Phenyl(dihydro)naphtalene-type lignanamides could be also the compounds under peaks **4**, **6** and **13**. The most polar compounds **4** and **6** showed the deprotonated molecular ions at *m/z* 609.1886 and 609.1887, respectively and were distinguishable with the other extract’s compounds for their content in octopamine, beyond tyramine, as polyamine moiety. In particular, both the [M − H]^−^ ions underwent water neutral loss (likely from the octopamine residue) providing a fragment ion at *m/z* 591.18 and presented the base peak ion at *m/z* 456.11 (Figure S11). This latter resembled that observed in compound **16**, in accordance with an arylnaphthalene lignandiamide core. Furthermore, TOF-MS/MS spectrum of compound **4** also displayed the fragment ion at *m/z* 472.1058, which could be due to the loss of the tyramine moiety, and further underwent water loss to yield the ion at *m/z* 454.0944 (Figure S11). The neutral loss of octopamine supplied for compound **6** directly the ion at *m/z* 456.1102, whose chemical feature likely resembled that of previously identified compound cannabisin I [27]. This latter was tentatively identified through TOF-MS and TOF-MS/MS spectra of compound **21**. Instead, compound **13** was likely a hydroxy derivative of *N*-caffeoyltyramine phenyl- dihydronaphtalene dimer. Its deprotonated molecular ion at *m/z* 613.2192 gave rise, through H_2_O neutral loss, to the base peak at *m/z* 595.2080, which in turn could lose 110.03 Da to provide the ion at *m/z* 485.1700, or could yield through 163.06 Da loss the ion at *m/z* 432.1446 (Figure S12). Both the ions at *m/z* 595.2080 and 432.1446 could undergo phenyldihydronaphthalene moiety cleavage providing the ions at *m/z* 475.1864 and 312.1227, respectively. All the other detected fragment ions could be from 110, 163 or 120 Da losses. Furthermore, a dimer of *N*-caffeoyloctopamine and *N*-caffeoyltyramine was hypothesized to be compound **26**, whose deprotonated molecular ion at *m/z* 611.2047 gave rise the abundant ions at *m/z* 314.1044 and 298.1088, attributable to caffeoyloctopamine, and caffeoyltyramine, respectively (Figure S13).

**Figure 4:**
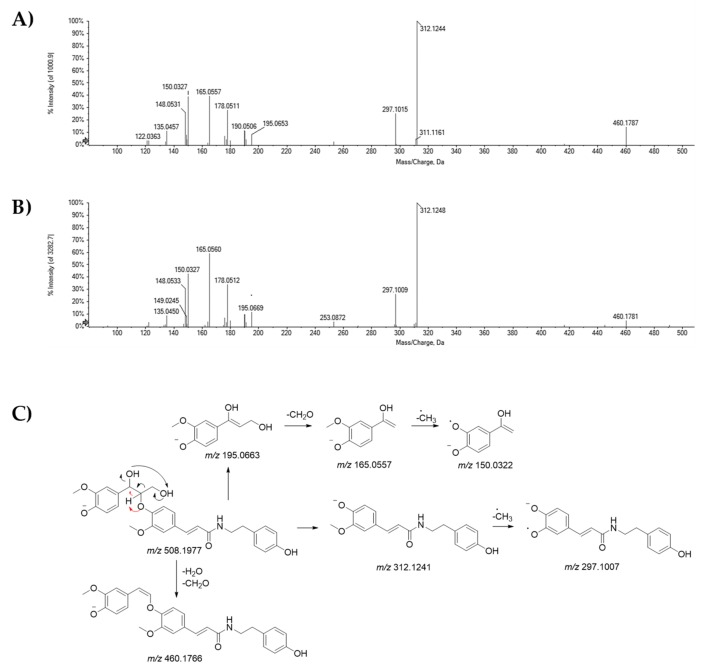
TOF-MS/MS spectra of compounds (**A**) **19** and (**B**) **20**, tentatively identified as cannabisin H isomers. The proposed fragmentation pathway of their [M − H]^−^ ion was reported (**C**); the theoretical *m/z* value is reported below each structure.

H_2_O and CH_2_O losses were detectable in TOF-MS/MS spectra of peaks related to compounds **19** and **20**, which showed the deprotonated molecular ions at *m/z* 508.1968 and 508.1990, respectively, likely corresponding *erythro* and *threo*-diastereoisomers of cannabisin H [42]. The fragment ions at *m/z* 312.1244 and 312.1248, could be formed following the cleavage of β-aryl ether moiety, whereas the further methyl radical loss yielded the ions at *m/z* 297.1015 and 297.1009. Diagnostic ions at *m/z* 195.0653(69), likely α-hydroxyconiferyl alcohols, and the deformylation products at *m/z* 165.0557(60), appeared to support our hypothesis (Figure 4). The *erythro* diastereomer, together with grossamide K, was isolated for the first time as a phenolic constituent of the bark of the kenaf (*Hibiscus cannabinu*s var. *Salvador*) [42].

**Figure 5:**
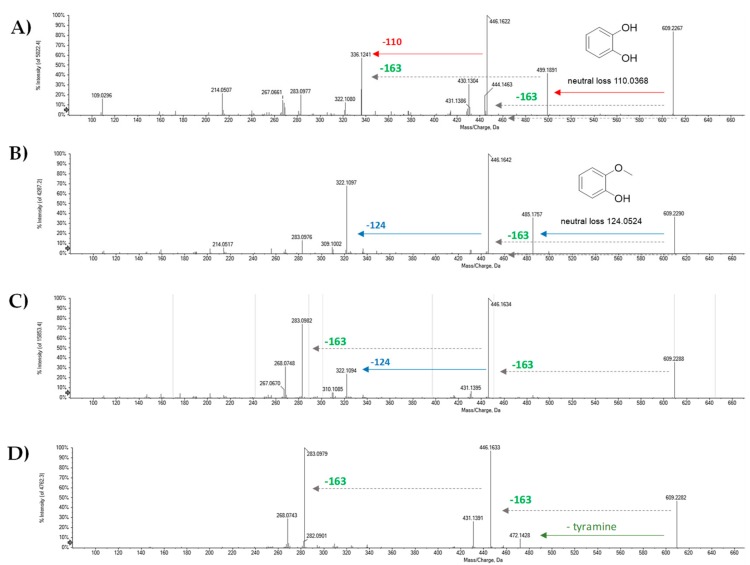
TOF-MS/MS spectra of compounds (**A**) **23**, (**B**) **24**, (**C**) **25**, and (**D**) **35**.

The deprotonated molecular ions of compounds **23–25** and **35** were in accordance with the molecular formula C_35_H_34_N_2_O_8_ and the occurrence of lignandiamides in which the hydroxycinnamoyl moieties were represented by caffeoyl and coniferyl alcohols, whereas tyramine constituted the amine part. In TOF-MS/MS spectra of compounds **23–25**, the base peak ion was at *m/z* 446.16, allowing us to confirm that the concurrent loss of isocyanic acid and *p*-hydroxystyrene could be advantageously observed as informative of tyramine presence. Moreover, other diagnostic fragment ions could be observed and differentiate the major bonding types encountered in hemp fruit lignandiamides. The aryldihydronaphtalene-type core likely characterized both compounds **23** and **24** which were distinguishable through fragment ions at *m/z* 499.1891 and 485.1757, respectively, which were in accordance with their relative catechol or guaiacol loss. This finding was in line monolignol cross-coupling with a caffeoyl-end-unit for compound **23** and a guaiacol-end unit for compound **24**. Based on previous observation, TOF-MS/MS spectrum of compound **35**, which appeared to fragment via a pathway similar to that of compound **28**, allowed us to hypothesize demethylgrossamide occurrence. In fact, also in this case, the [M − H]^−^ ion underwent tyramine neutral loss with the genesis of the ion at *m/z* 472.1428, whereas the loss of 163.06 Da gave the most abundant ion at *m/z* 446.1633, which in turn lost a methyl radical, providing the radical anion at *m/z* 431.1391. Furthermore, as already observed in compound **28**, the loss of two 163.06 Da units could occur giving the ion at *m/z* 283.0979. This latter, which represented the base peak, also furnished the radical ion at *m/z* 268.0743 through methyl radical loss (Figure 5). UV-DAD spectra were in accordance with tentatively assigned lignandiamide skeleton, and represented a useful tool to unravel the lignan nucleus of compound **25**, which was supposed to belong to aryldihydronaphtalene class [26].

Based on previous MS observations, and UV-DAD spectra, compounds **27**, and **29**, whose pseudomolecular ions were in accordance with the C_36_H_36_N_2_O_8_ molecular formula, and showing the base peak at *m/z* 460.1799(1803), and its demethylated radical ion at *m/z* 445.1569(1), were suggested to be aryldihydronaphtalene-type lignanamide isomers, whereas compound **37** was tentatively identified as cannabisin F, and compound **39** was putatively assigned as grossamide (Figure S14). This latter compound, whose MS/MS fragmentation pattern in positive ion mode was previously reported [43], was found to exert anti-neuroinflammatory action, being able to inhibit the secretion of pro-inflammatory mediators (e.g., IL-6, and TNF-α), reducing LPS-mediated IL-6 and TNF-α mRNA levels [44]. Furthermore, neuroprotection by cannabisin F was ascertained against LPS-induced inflammatory response and oxidative stress in BV2 microglia cells [21].

Grossamide K, a phenylcoumaran-type lignanamide with previously reported antimelanogenic activity [45], was tentatively identified based on TOF-MS and TOF-MS/MS spectra related to peak **30**. The deprotonated molecular ion at *m/z* 490.1875 provided the fragment ions at *m/z* 472.1769 and 460.1769, following H_2_O and formaldehyde neutral losses. Charge-driven CH_2_O loss could be initiated when phenoxide ion abstracted the proton from aliphatic OH function. Both the ions underwent methyl radical loss to achieve ions at *m/z* 457.1541 and 445.1529, respectively, which, in turn, gave diradical anions at *m/z* 442.1300 (base peak) and 430.1300. This latter could provide the fragment ion at *m/z* 297.1125 through CO-Cα cleavage, or the ion at *m/z* 338.1027 by dehydrogenation and hydroxystirene loss. Methyl radical and CO losses were further detected (Figure S15).

Finally, compounds **31** and **34** were tentatively assigned as cannabisin E isomers based on their deprotonated molecular ion at *m/z* 641.2516, which provided the ion at *m/z* 489.2053(64) through 4-hydroxy-3-methoxy benzaldehyde loss. The detection of the ion at *m/z* 151.0404(6) as base peak, attributable to 4-formyl-2-methoxyphenolate, seemed to confirm the hypothesis (Figure S16).

### 2.3. HRMS Analysis: Flavonol Glycosides

Compounds **3**, **7**, **9**, **10**, **12**, **14** and **18** were putatively flavonol glycosides (Figures S17–S20, Table 3), whose presence was previously identified as bioactive components of hemp seeds, and its cold-pressed oil derived product [24]. In particular, collision-induced dissociation of the [M − H]^−^ ion at *m/z* 433.0777 for compound **3**, gave radical aglycone ion [aglycone − H]^−^ at *m/z* 300.0273 and its aglycone ion at *m/z* 301.0362, which were consistent with quercetin and the loss of a pentose moiety (−132.0415 Da). The presence of the pentose moiety was further suggested for compounds **9** and **10**, whose aglycone moiety was kaempferol, as suggested by the [aglycone − H]^–^ and aglycone radical ion at *m/z* 285.0406(08) and 284.0328(29), as well as the ions at *m/z* 255.0299(303) and 227.0352(1), which could come from the [aglycone − H]^•−^ ion. The loss of deoxyhexose moiety was shared by compounds **7**, **12** and **14**. Quercetin was identified as aglycone residue in compound **7**, whereas compounds **12** and **14** were kaempferol deoxyhexoside isomers. Finally, the detected loss of acetic acid moiety in the TOF-MS/MS spectrum of compound **18** as well as the occurrence of [aglycone–H]^–^, [aglycone − H]^•−^ and [aglycone − 2H]^•−^ at *m/z* 301.0361; 300.0278 and 299.0194, were in accordance with quercetin-7-O-acetyldeoxyhexose [46,47]. Quantifying flavonoid content as peak area percentage, it was found that the compounds constituted only the 2.8%, whereas lignanamides were the most representative compounds with the 79.0%. Compounds deriving from the coupling of two phenylamides, both preserving the amine moiety (e.g., metabolites showing the [M − H]^−^ at *m/z* 593, 595, 609, 623, 641), were the most abundant and they were present in comparable amount (Figure S21).

### 2.4. LnHS Inhibits Cell Survival of Glioblastoma U-87 Cell Line but not of HF

**Figure 6:**
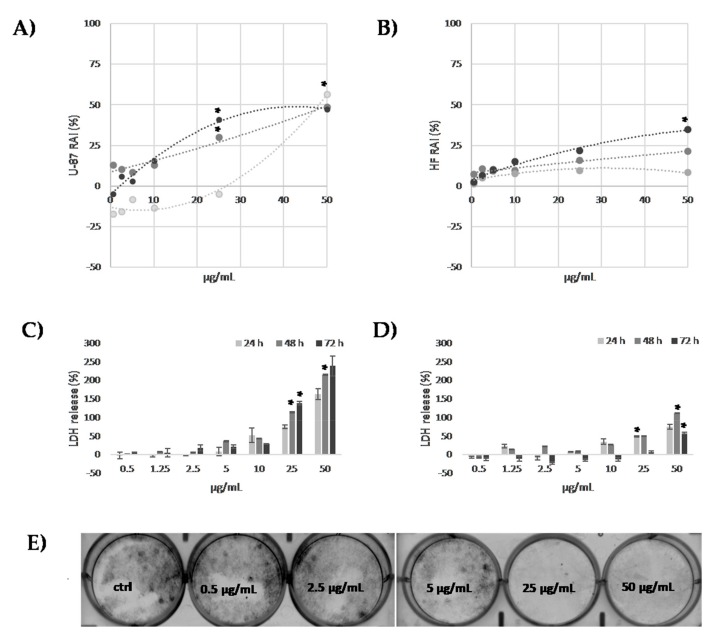
Cell viability of U-87 and human HF cell (**A** and **B**, respectively) was assessed by MTT assay after 24, 48 and 72 h of exposure. Data from LDH release assay at 24, 48 and 72 h exposure times were in panels **C** (U-87 cells) and **D** (HF cells). Values are the mean ± SE of two independent experiments performed in triplicate. *p < 0.05 vs. untreated cells. (**E**) Representative images from colony forming efficiency of U-87 cells grown in presence of LnHS or vehicle control for ten days; the experiment was performed in duplicate.

As some of compounds in LnHF were reported to exert neuroprotective and anti-neuroinflammatory effects [20,21], or also to be able to induce dramatic morphological changes and autophagic cell death [11], the effects of the treatment of LnHS hempseed mixture on primary glioblastoma cell lines was investigated. Indeed, a prior analysis of the effects of LnHS was carried out on undifferentiated SH-SY5Y cells. It was evidenced that LnHS at dose levels higher than 25 μg/mL, induced clear cell morphological changes as cells shrunk and loss cell adhesion (Figure S2). Thus, in order to better understand and define a preliminary LnHS-cell death inducing mechanism, the effects on cytotoxicity was further assessed on U-87 glioblastoma cells, which are characterized by a rapid migration, and a highest invasion capacity [48]. Human Fibroblast (HF) cells were used in order to evaluate LnHS effects on non-tumorigenic cells. MTT assay was performed to assess the effects of LnHS on cell viability, at seven dose levels (0.5, 1.25, 2.5, 5, 10, 25, 50 µg/mL), and at three exposure times (24 h, 48 h and 72 h). Data acquired showed that LnHS exerted toxic effects on U-87 cells. Indeed, U-87 cell viability was strongly compromised at the highest LnHS tested dose, at each exposure time considered (Figure 6). The dose level 25 µg/mL significantly affects the U-87 viability after 48 and 72 h of incubation, *p* < 0.05 (Figure 6A). LnHS did not show relevant toxic effects on HF cells, and only the highest dose (50 µg/mL) appeared to induce, after the longer 72 h exposure time, cell viability decrease (Figure 6B). In addition, the quantification of lactate dehydrogenase levels, considered an indicator of cell damage for necrotic cell death [49], evidenced that LnHS 25 µg/mL and 50 µg/mL dose levels exerted a time-dependent percentage increase of LDH release (Figure 6C,D). Furthermore, the colony formation assay [50] showed that LnHS-treated U-87 cells did not retain the capacity to produce colonies (Figure 6E), when LnHS dose exceeded only 5 µg/mL.

### 2.5. LnHS Induced Genomic DNA Damage in U-87 Cell Line but not in HF

**Figure 7:**
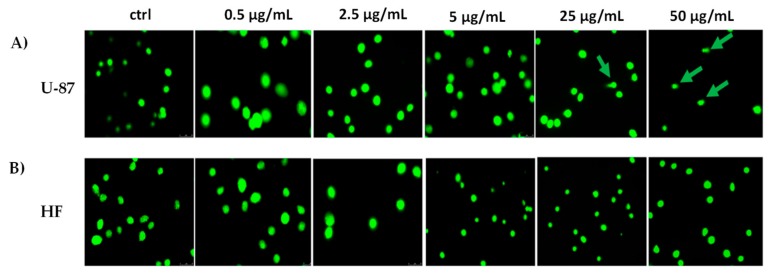
Representative images of U-87 and HF cells treated with different LnHS doses and subjected to the comet assay (panels **A** and **B** respectively). ctrl: untreated cells; green arrows indicate comets with a tail. Experiments were performed in duplicate.

In order to verify if the observed cytotoxicity was the result of LnHS ability to induce a genotoxic damage, comet assay was performed on U-87 and HF cells, after 24 h of exposure to the hempseed mixture at 0.5, 2.5, 5, 25 and 50 μg/mL concentration levels (Figure 7). U-87 cells showed DNA damage when treated with LnHS 25 and 50 μg/mL (Figure 7A), whereas no genotoxic effects was detectable in human fibroblast cells.

### 2.6. LnHS Inhibited Cell Migration of U-87 Cell Line

**Figure 8:**
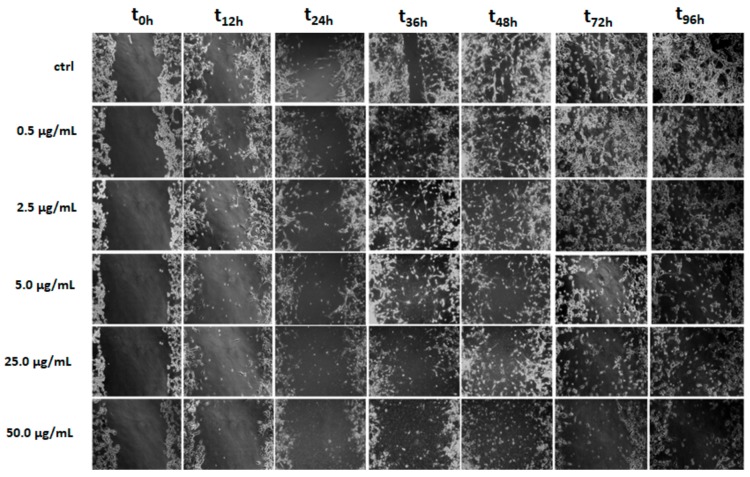
U-87 cells underwent a scraped wound and were then treated with different LnHS doses (0.5, 2.5, 5, 25 and 50 μg/mL). Cells were photographed immediately following the scratch (0 h), after 12, 24, 36, 48, 72 and 96 h. Untreated cells (ctrl), were used as a control. Representative figures are shown from one of two independent experiments.

As invasiveness is one of the pathophysiological features of human malignant gliomas [51], the effects of LnHS on the U-87 cells migration, in comparison to HF, were tested through wound healing assay. Both the cell types were subjected to scraped wounds: U-87 cells were treated with five doses (0.5, 2.5, 5, 25 and 50 µg/mL), whereas HF cells with three LnHS doses (5, 25 and 50 µg/mL). Although LnHS significantly inhibited the migration of both cell types at the highest dose, U-87 appeared more sensitive to LnHS (Figure 8). In fact, already at a dose of 5 µg/mL, the hempseed fraction inhibited the migration of the U-87 cells; the maximum effect was evident at 50 µg/mL, where the cell toxicity is also evident.

### 2.7. LnHS did not Modify Sirtuins and Cytokines Expression in both U-87 and HF Cells

To verify LnHS ability to display cytotoxicity, interfering oxidative and inflammation processes, the expression of Sirt1 and Sirt2, as well as of some pro-inflammatory cytokines involved, was evaluated. In this context, cannabisin F was recently found to act as a modulator of SIRT1, whose activation is considered beneficial in attenuating neuro-inflammation and damage due to oxidative stress [21]. Thus, both U-87 and HF cells were treated with three LnHS doses (2.5, 25 and 50 μg/mL) for 24 and 48 h, and the expression level of Sirt1, Sirt2, IL-6 and IL-10 was assessed at mRNA level. Indeed, no effects were found on these markers in both the cell types (Figure S22).

### 2.8. LnHS Blocks Autophagy While Inducing Apoptosis in U-87 Cells

**Figure 9:**
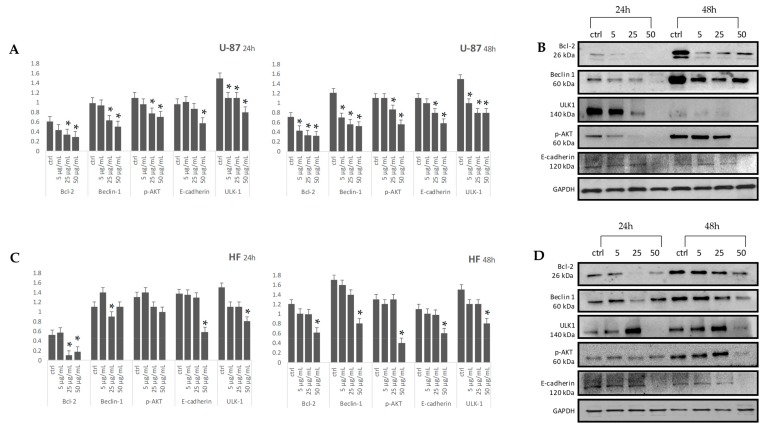
Levels of Bcl-2, Beclin-1, p-AKT, E-cadherin and ULK-1 in U-87 and HF cell types were detected using western blotting with respective antibodies. Graphical representation of pixel quantization of Bcl-2, Beclin-1, p-AKT, E-cadherin, and ULK-1 normalized to GAPDH (panels **A**, **C**). The intensities of signals were expressed as arbitrary units. *p < 0.05 vs. untreated cells (ctrl). Representative western blotting image of Bcl-2, Beclin-1, p-AKT, E-cadherin, ULK-1 and GAPDH are in panels **B** (U-87 cell type), and **D** (HF cell type).

Autophagic cell death was hypothesized to be the mechanism through which cannabisin B exerts antiproliferative activity in human hepatocarcinoma HepG2 cells [11]. Autophagy is a cell catabolic program, and it is triggered following nutrient starvation, and requires for its initiation ULK kinases [52]. In particular, ULK-1 phosphorylates Beclin-1 is a multi-domain protein, which exerts a dual role in autophagy and apoptosis cellular processes [53]. LnHS treatment at dose levels equal to 5, 25 and 50 μg/mL, at 24 and 48 h exposure times, promoted the down-regulation of Beclin 1 and ULK-1 in U-87 cells, while in fibroblasts, both the enzymes were down-regulated only by 50 μg/mL dose (Figure 9). Simultaneously, it was found that Bcl-2 was also inhibited at the same incubation times and LnHS doses (Figure 9). Altogether, these results indicated a strong down-regulation of autophagy process in U-87 cells. These two processes are accompanied by reduction in AKT phosphorylation. E-cadherin expression was affected at 50 mL at 24 and 48 h in both cell types. This finding is in line with a potential double-edge behavior of LnHS, which, according to autophagy occurrence, at low doses appears to maintain E-cadherin expression, while, on the contrary, at the highest dose, LnHS may determine a cell-cell adhesion injury by the E-cadherin loss (Figure 9).

## 3. Materials and Methods

### 3.1. Materials

All the solvents used for extraction and fractionation purposes, acetonitrile (LC–MS grade), formic acid (98%, for mass spectrometry) were purchased from Sigma-Aldrich (Buchs, Switzerland). Cell culture media and reagents for cytotoxicity testing were purchased from Invitrogen (Paisley, Scotland, UK), except MTT [3-(4,5-dimethyl-2-thiazolyl)-2,5-diphenyl-2H-tetrazolium bromide] which was from Sigma-Aldrich Chemie GmbH (Schnelldorf, Germany).

### 3.2. Plant Extraction and Fractionation

One hundred g of commercial hemp seeds (*Hanf* & *Natur* product, by Bioland, a certified company for the cultivation of ecological and sustainable hemp located in Lindlar, Germany), after mechanical reduction, underwent ultrasound assisted maceration (UAM) using sequentially *n*-hexane and ethanol as extracting solvents. The drug/solvent ratio used was 1:5. Ultrasound (mechanic waves able to propagate through an elastic medium) maceration cycles were in total 4 and performed using a Bransonic^TM^ M3800-E device (Branson Ultrasonics^TM^, Danbury, CT, USA) operating in sweep-frequency mode at 40 kHz. The duration of each ultrasound cycle was 30 min. The alcoholic fraction (4.4 g) was then chromatographed through SiO_2_ column chromatography (h 16 cm, Ø 2.0 cm), eluting with chloroform, ethyl acetate and methanol providing three subfractions. The methanol extract, named LnHS, underwent chemical investigation by means of UHPLC-HR-MS/MS and HPLC-UV-DAD analyses and cytotoxicity assessment towards U-87 human glioblastoma cells. The extraction/fractionation scheme is depicted in Figure S1.

### 3.3. UHPLC-ESI-QqTOF-MS/MS and HPLC-UV-DAD Analyses

LnHS was chemically characterized by ultrahigh-performance liquid chromatography coupled with high-resolution mass spectrometry (UHPLC-HRMS) techniques. A NEXERA UHPLC system (Shimadzu, Tokyo, Japan) was used with a Luna^®^ Omega Polar C-18 columns (1.6 μm particle size, 50 × 2.1 mm, Phenomenex, Torrance, CA, USA). Separation was achieved with a linear gradient of water (A) and acetonitrile (B), both with 0.1% formic acid: 0–3 min, 2→12.5% B; 3–12.5 min, 12.5→30% B; 12.5–17.5 min, 30→45% B; 17.5–20 min, 45→75% B; held at 75% B for other 2 min; 22–23 min, 75→98% B. The mobile phase composition was maintained at 98% B for another 1 min, then returned to the starting conditions and allowed to re-equilibrate for 1 min. The total analysis time was 26 min, the flow rate was 0.5 mL/min, and the injection volume was 2.0 µL.

MS analysis was performed using a hybrid Q-TOF MS instrument, the AB SCIEX Triple TOF^®^ 4600 (AB Sciex, Concord, ON, Canada), equipped with a DuoSpray^TM^ ion source (consisting of both electrospray ionization (ESI) and atmospheric pressure chemical ionization (APCI) probes), which was operated in the negative ESI mode. The APCI probe was used for automated mass calibration using the Calibrant Delivery System (CDS). The CDS injects a calibration solution matching the polarity of ionization and calibrates the mass axis of the TripleTOF^®^ system in all scan functions used (MS and/or MS/MS). The Q-TOF HRMS method, which combines TOF-MS and MS/MS with Information Dependent Acquisition (IDA) for identifying non-targeted and unexpected compounds, consisted of a full scan TOF survey (dwell time 100 ms, 100–1000 Da) and a maximum number of eight IDA MS/MS scans (dwell time 50 ms, 80–850 Da). The MS parameters were as follows: curtain gas (CUR) 35 psi, nebulizer gas (GS 1) 60 psi, heated gas (GS 2) 60 psi, ion spray voltage (ISVF) 4.5 kV, interface heater temperature (TEM) 600 °C, declustering potential (DP) −80 V. Collision Energy (CE) applied was −45 V with a collision energy spread (CES) of 15 V. The instrument was controlled by Analyst^®^ TF 1.7 software, while data processing was carried out using PeakView^®^ software version 2.2. Hydrogen/deuterium exchange experiments were carried out on pure *N*-caffeoyltyramine as previously described [54]. Furthermore, in order to achieve UV-DAD information of each peak, separation was also performed by using a 1260 Infinity II LC System (Agilent, Santa Clara, CA, USA) equipped with an Agilent G711A quaternary pump and a WR G7115A diode array detector.

### 3.4. Cell Culture and Cytotoxicity Assessment

The human glioblastoma U-87 cell line and human fibroblasts (HF) were kindly provided by the Bank of Human and Animal Continuous Cell Lines-CEINGE Biotecnologie Avanzate – Napoli - Italy. The U-87 cell line was cultured in Dulbecco’s medium (DMEM) supplemented with 10% of heat-inactivated Fetal Bovine Serum (FBS) and 1% l-glutamine (Sigma-Aldrich, St. Louis. MO, USA); HF were cultured in cultured in Dulbecco’s medium (DMEM) supplemented with 20% of heat-inactivated Fetal Bovine Serum (FBS) and 1% l-glutamine (Sigma-Aldrich). Both cells were grown in a 5% CO_2_ humidified incubator, at 37 °C. For treatments, the cells were incubated with LnHS at different concentrations in serum-free fresh medium for different incubation times.

#### 3.4.1. MTT Cell Viability Assay

U-87 and HF cells were seeded in 10% FBS-containing medium in a 96-well plate at the density of 3 × 10^3^ cells/well. The day after, the cells were treated as above described. Cell viability was evaluated by the 3-(4,5-dimethylthiazol-2-yl)-2,5-diphenyltetrazolium bromide (MTT; Sigma-Aldrich) assay. The next day, cells were treated as above described and, after incubation times, the assay was performed as previously described [55].

#### 3.4.2. LDH Release Assay

The lactate dehydrogenase (LDH) leakage assay is a colorimetric test useful for quantifying cell death and lysis through the measurement of LDH released from the cytosol of damaged cells, which was into supernatant. The test was carried out as previously reported [56].

#### 3.4.3. Colony Forming Assay

For colony forming assays, U-87 cells (1 × 10^3^) were seeded per well of a 6-well plate and maintained for 10 days with medium changed every other day. Colonies were stained using crystal violet (Sigma-Aldrich) at room temperature for 20 min and washed repeatedly in water. Colonies were counted manually using a light microscope as previously described [57].

#### 3.4.4. Comet Assay

DNA breakage was evaluated using a comet assay kit (Trevigen, Gaithersburg, MD, USA). Three independently reproduced experiments were performed. To determine DNA strand breakage, U-87 cells and HF were plated at 1 × 10^5^ cells/mL in a 24-well plate and left to attach for 24 h. The next day, cells were treated with LnHS (0.2, 2.5, 5, 25, 50 μg/mL) for 24 h. Comet slides were stained with diluted SYBR green and examined under an automated robotic epifluorescence microscope with an excitation filter (510 to 550 nm). One hundred cells were analyzed per slide. Experiments were performed in duplicate. Untreated cells served as negative controls.

#### 3.4.5. Wound Healing Assay

U-87 cells and HF were seeded at a density of 3 × 10^5^ cells in a 6-well plate in complete culture media and grown to confluence. The day after, cells were treated with 4 μg/mL of mitomycin (Sigma-Aldrich) for 2 h to inhibit cell proliferation and then a wound was inflicted using a tip. After washing with phosphate-buffered saline (PBS), cells were incubated as above mentioned in comparison to untreated cells. The scratch wound was observed and photographed at different time points using an inverted-phase-contrast microscope (TS100 fluorescence microscope and video camera, Nikon, Tokyo, Japan). Three measurements per scratch were performed (two replicates/condition, experiments performed in duplicate).

#### 3.4.6. RNA Extraction and Real Time Quantitative PCR

U-87 and HF cells, after 12 h starvation, were treated in 0% FBS medium as above described. After incubation, total RNA was isolated from using TRIzol (Invitrogen, Carlsbad, CA, USA) and Real-time PCR was performed as previously described using GAPDH as housekeeping gene [58]. The primers for Sirt1, Sirt2, IL-6, IL-10 and GAPDH are available on request. The experiments were performed two times in triplicate.

#### 3.4.7. Preparation of Cell Extracts and Western Blotting Analysis

U-87 cells and HF cells, after 12-h starvation, were treated in 0% FBS medium as above described. After incubation, total proteins, extracted in RIPA buffer, were quantified by Bradford’s method (Bio-Rad, Hercules, CA, USA). Total proteins were subjected to electrophoresis and transferred to PVDF membranes; the membranes were incubated with the following antibodies according to the manufacturer’s instructions: E-cadherin and GAPDH (Santa Cruz Biotechnology, Dallas, TX, USA), p-AKT, ULK-1, Bcl-2, Beclin 1 (Cell Signaling Technology, ZA Leiden, Netherlands). The blots were developed by ECL (Amersham Biosciences, Piscataway, NJ, USA) and analyzed by densitometry as previously described [58]. Each sample was tested three times in duplicate.

### 3.5. Statistical Analysis

Experiments were performed three times with replicate samples, except where otherwise indicated. Data are expressed as mean ± SD (standard deviation). The means were compared using analysis of variance (ANOVA) plus Bonferroni’s *t*-test. A *p*-value of < 0.05 was considered to indicate a statistically significant result.

## 4. Conclusions

The recovery of bioactive compounds (pure or in their mixture form) from hempseed meal could be the driving force for developing new hemp seed-based goods, in which the processing of hemp fruits waste is valuable for fully exploiting the innumerable advantages of this crop [59,60]. In this context, high resolution negative tandem mass spectrometric techniques could be a useful tool in the structure elucidation of hemp seeds compounds such as phenylamides and lignanamides.

The main phenylamides in hemp seeds are hydroxycinnamoyl amides in which the amine moiety was octopamine or tyramine. They are readily differentiable based on their TOF-MS^2^ spectra as the octopamine conjugate promptly lost a water molecule and the base peak corresponds to a hydrocinnamoyl moiety. Caffeoyl and feruloyl-derived phenyldihydronaphthalene lignanamides are distinguishable by a characteristic loss of 110 and 124 Da, whereas phenylcoumaran lignandiamide were characterized by a first direct tyramine moiety. Moreover, isocyanic acid (HNCO) + *p*-hydroxystyrene represent a common neutral loss for all the identified lignanamides. The presence of an α,β-unsaturated function favors a facile CO-Cα cleavage, and low fragment ions give additional structural information on the investigated lignanamides. Cytotoxicity assessments of LnHS on the U-87 glioblastoma cell line and on human fibroblasts provided new insight into the molecular effects of this lignanamide extract. Indeed, even if further studies are necessary, our data strongly suggest that LnSH negatively and specifically regulates U-87 cell line survival and migration then reinforcing the need to fully analyze its biochemical behavior, and lignanamides purified therefrom. Indeed, the compounds’ purification will be addressed in order to deeply investigate their quantitation in hemp seeds products from the different cultivars available on the market, as well as to achieve a clearer picture of their already promising anti-cancer activity. In this context, the ability of LnHS (and pure compounds therefrom) to cross the blood brain barrier (BBB) is aimed to be promptly pursued. This is in line with several recent evidences which highlight the ability of dietary polyphenols, and their known physiologically relevant metabolites, to enter the brain endothelium, cross the BBB and to impact brain health and cognition [61,62,63]. The BBB-crossing feature in phenylamides and lignanamides could be improved by the intrinsic amide function occurrence. In fact, recent studies showed that the introduction of an amide function was a strategy to enhance BBB transport of antineoplastic drug [64], whereas the development of *N*-acetylcysteine amide (NACA) preserved *N*-acetylcysteine antioxidant ability improving its permeability through cell membranes [65]. Furthermore, considering the identified compounds mainly as polyamine derivatives, it was recently shown that tyramine analogue in *Gingko biloba* extract were identified among compounds able to cross BBB [66].

## Figures and Tables

**Table 1:** TOF-MS and TOF-MS/MS of tentatively identified phenylamides in the investigated hempseed fraction. RT = Retention Time; RDB = Ring Double Bond equivalent value. Compounds were numbered based on their RT in the whole LnHS chromatogram.

	RT (min)	Tentative Assignment	Formula	[M − H]^−^ Calc. (*m/z*)	[M − H]^−^ Found (*m/z*)	Error (ppm)	RDB	MS/MS Fragment Ions (*m/z*) and Relative Intensity (%)
**1**	4.190	N-caffeoyloctopamine	C_17_H_17_NO_5_	314.1034	314.1039	1.6	10	296.0910(28.2); 269.0818(5.5); 254.0813(4.2); 161.0244(86.7); 160.0396(15.8); 135.0455(100); 134.0609(19.5); 134.0373(22.5); 133.0302(47.1); 132.0217(8.3); 123.0449(1.1); 107.0505(13.9); 93.0350(7.3)
**2**	5.141	*N*-p-coumaroyloctopamine	C_17_H_17_NO_4_	298.1085	298.1086	0.4	10	280.0981(11.7); 160.0408(12.3); 145.0293(97.5); 134.0607(15.9); 133.0531(18.0); 119.0502(100); 117.0347(84.2); 93.0342(7.5)
**5**	6.426	*N*-caffeoyltyramine	C_17_H_17_NO_4_	298.1085	298.1090 334.0871 [M + Cl]^−^	1.7	10	298.1102(19.6); 256.0974(2.8); 190.0518(16.2); 178.0520(20.6); 161.0251(11.3); 148.0536(18.5); 147.0456(10.5); 136.0775(6.0); 135.0459(100); 134.0381(18.6); 133.0301(8.1); 107.0508(7.8)
**8**	7.151	*N*-feruloyltyramine	C_18_H_19_NO_4_	312.1241	312.1243	0.5	10	312.1247(13.0); 297.1015(7.6); 190.0510(61.1); 178.0513(63.9); 176.0354(13.9); 148.0530(100); 147.0448(24.9); 135.0452(35.2); 134.0373(8.7)
**11**	7.755	*N*-p-coumaroyltyramine	C_17_H_17_NO_4_	282.1136	282.1140	1.5	10	282.1140(4.9); 174.0559(7.8); 162.0558(9.4); 145.0294(4.3); 132.0580(7.4); 119.0503(100); 117.0341(9.4)
**17**	8.387	*N*-feruloyltyramine	C_18_H_19_NO_4_	312.1238	312.1241	−1.1	10	312.1254(6.8); 297.1014(6.1); 190.0513(65.5); 178.0513(57.4); 176.0354(14.5); 148.0535(100); 147.0456(30.2); 135.0456(37.2); 134.0376(11.8)
**33**	12.209	tri-p-coumaroylspermidine	C_34_H_37_N_3_O_6_	582.2610	582.2640 618.2418 [M + Cl]^−^ 628.2695 [M + FA]^−^	5.2	18	582.2662(21.2); 462.2064(55.7); 436.2266(7.3); 342.1472(100); 316.1675(27.1); 299.1406(9.9); 145.0300(20.7); 119.0508(58.5)

**Table 2:** TOF-MS and TOF-MS/MS of tentatively identified lignanamides in the investigated hempseed fraction. RT = Retention Time; RDB = Ring Double Bond equivalent value. Compounds were numbered based on their RT in the whole LnHS chromatogram.

	RT (min)	Tentative Assignment	Formula	[M − H]^−^ calc. (*m/z*)	[M − H]^−^ Found (*m/z*)	Error (ppm)	RDB	MS/MS Fragment Ions (*m/z*) and Relative Intensity (%)
**4**	6.204	*N*-caffeoyltyramine/ *N*-caffeoyloctopamine dimer 1	C_34_H_30_N_2_O_9_	609.1879	609.1886	1.2	21	609.1909(63.7); 591.1788(7.0); 472.1058(14.2); 456.1103(100); 454.0944(8.2); 428.1152(14.4)
**6**	6.766	*N*-caffeoyltyramine/ *N*-caffeoyloctopamine dimer 2	C_34_H_30_N_2_O_9_	609.1879	609.1887	1.4	21	609.1897(5.5); 591.1799(18.4); 456.1102(100)
**13**	8.031	*N*-caffeoyltyramine dimer hydroxy derivative	C_34_H_34_N_2_O_9_	613.2192	613.2192	0.1	19	613.2192(56.0); 595.2080(82.8); 503.1798(9.9); 485.1700(35.8); 475.1864(44.5); 450.1552(5.1); 432.1146(72.5); 322.1071(84.9); 312.1227(100); 298.1065(5.2); 269.0809(65.2); 229.0500(30.0); 159.0448(10.1); 137.0248(85.3)
**15**	8.150	Cannabisin B	C_34_H_32_N_2_O_8_	595.2086	595.2095	1.5	20	595.2081(28.2); 485.1712(29.0); 432.1447(56.7); 430.1280 (7.5); 322.1076(60.3); 269.0815(100); 214.0507(7.4); 202.0513(3.9); 159.0450(7.9); 109.0299(7.5)
**16**	8.268	Cannabisin A	C_34_H_30_N_2_O_8_	593.1929	593.1941	2.0	25	593.1958(92.5); 575.1850(4.5); 473.1373(4.4); 456.1110(100); 430.1313(59.9); 428.1151(5.3); 349.0595(4.8); 336.0520(3.9); 322.0723(10.2); 293.0457(11.5); 263.0349(6.1)
**19**	8.840	Cannabisin H isomer 1	C_28_H_31_NO_8_	508.1977	508.1968	1.8	14	460.1787(17.9); 312.1244(100); 311.1161(5.9); 297.1015(22.1); 195.0653 (6.2); 190.0506(8.1); 178.0511(23); 165.0557(25.4); 150.0327(19.9); 148.0531(22.8); 135.0457(6.2); 122.0363(5.1)
**20**	9.019	Cannabisin H isomer 2	C_28_H_31_NO_8_	508.1977	508.1990	2.6	14	460.1781(6.5); 312.1248(100); 297.1009(26.3); 195.0669 (11.5); 190.0661(11.8); 178.0512(33.7); 165.0560(56.8); 150.0327(43.5); 148.0533(29.8); 135.0450(6.9)
**21**	9.118	Cannabisin I	C_26_H_19_NO_7_	456.1089	456.1095	1.4	18	456.115 (100); 428.1167(6.0); 414.0996(16.1); 348.0529(25.2); 336.0527 (7.0); 320.0593 (7.0); 306.0416 (7.0); 293.0464 (13.1)
**22**	9.213	Cannabisin B isomer	C_34_H_32_N_2_O_8_	595.2086	595.2095	1.5	20	595.2089(33.6); 485.1708(22.8); 432.1449(53.7); 338.1032(1.2); 322.1077 (68.5); 295.0592(10.6); 269.0814(100); 254.0576(11.3); 214.0498(7.8); 202.0506(6.5); 159.0456(9.7); 147.0455(6.3); 109.0300(8.4)
**23**	9.622	Cannabisin C	C_35_H_34_N_2_O_8_	609.2242	609.2249	1.1	20	609.2267(83.6); 499.1891(41.8); 446.1622(100); 444.1463(18.2); 430.1304(30.9); 336.1241(57.4); 322.1080(12.7); 283.0977(20.3); 267.0661(14.5); 214.0507(21.7); 109.0296(15.9)
**24**	9.961	Cannabisin C isomer	C_35_H_34_N_2_O_8_	609.2242	609.2254	2.1	20	609.2290(36.5); 485.1757(35.7); 446.1642(100); 322.1097(67.8); 309.1002(6.1); 283.0976(12.8); 214.0517(5.6)
**25**	10.210	*N*-caffeoyltyramine/ *N*-feruloyltyramine dimer1	C_35_H_34_N_2_O_8_	609.2242	609.2255	1.9	20	609.2288(35.9); 446.1634(100); 431.1395(6.6); 322.1094(24.1); 310.1085(5.1); 283.0982(74.1); 268.0748(31.2); 267.0670(8.0)
**26**	10.407	*N*-caffeoyltyramine/ *N*-caffeoyloctopamine dimer2	C_34_H_32_N_2_O_9_	611.2035	611.2047	2	20	611.2067(1.5); 593.1973(1.2); 567.2161(1.3); 430.1310(3.0); 402.1355(1.1); 314.1044(31.8); 312.0881(6.2); 298.1088(100); 296.0931(58.5); 161.0241(8.4); 135.0450(13.7)
**27**	10.844	Cannabisin D	C_36_H_36_N_2_O_8_	623.2399	623.2414	2.4	20	623.2458(40.0); 460.1799(100); 458.1648(23.3); 445.1569(25.0); 444.1490(51.6); 443.1409(9.4); 350.1411(5.6); 336.1255(23.4); 322.1097(8.7); 283.0983(34.5); 282.0904(11.6); 267.0669(11.6); 214.0517(5.7)
**28**	11.023	3,3′-didemethylgrossamide	C_34_H_32_N_2_O_8_	595.2086	595.2110	4.0	20	595.2102(11.3); 458.1262(17.2); 432.1468(48.4); 338.1037(7.5); 295.0616(10.6); 269.0828(100); 147.0462(8.9); 121.0306(10.5)
**29**	11.319	Cannabisin D isomer	C_36_H_36_N_2_O_8_	623.2399	623.2408	1.5	20	623.2459(19.8); 499.1914(6.4); 487.1915(5.3); 460.1803(100); 445.1561(15.8); 339.1126(5.4); 336.1255(28.5); 322.1094(22.7); 283.0983(9.7); 216.0673(5.8); 123.0456(5.2)
**30**	11.676	Grossamide K	C_28_H_29_NO_7_	490.1871	490.1875	0.8	15	472.1769(38.8); 460.1769(24.7); 457.1541(69.2); 445.1529(22.2); 442.1300(100); 440.1497(7.7); 430.1300(27.2); 414.1348(19.9); 338.1027(13.5); 323.0799(5.4); 308.1041(7.6); 297.1125(18.9); 293.0809(7.7); 283.0967(7.7); 276.0795(5.7); 267.0661(9.5)
**31**	11.715	Cannabisin E	C_36_H_38_N_2_O_9_	641.2505	641.2516	1.5	19	641.2540(10.4); 623.2435(16.7); 591.2164(6.6); 489.2053(35.3); 471.1576(6.3); 460.1779(27.4); 432.1824(18.4); 431.1986(43.3); 428.1508(7.4); 369.1455(8.5); 337.1191(12.9); 328.1191(33.8); 312.1241(93.4); 311.1396(21.4); 297.1003(10.2); 254.1183(5.8); 191.0349(6.7); 178.0508(8.0); 165.0555(14.8); 151.0404(100); 136.0170(34.8)
**32**	11.958	3,3′-demethyl-heliotropamide	C_34_H_32_N_2_O_8_	595.2086	595.2098	2.0	20	298.1083(100); 178.0503(2.8); 135.0444(5.7)
**34**	12.073	Cannabisin E isomer	C_36_H_38_N_2_O_9_	641.2505	641.2516	1.8	19	641.2555(12.3); 623.2452(10.6); 489.2064(42.0); 460.1795(45.6); 432.1839(12.1); 431.2002(37.9); 428.1527(6.0); 369.1470(9.8); 337.1203(15.8); 328.1202(13.5); 312.1253(85.8); 311.1405(20.9); 297.1015(8.4); 254.1193(5.8); 178.0510(7.4); 151.0406(100); 136.0170(36.8)
**35**	12.305	Demethylgrossamide	C_35_H_34_N_2_O_8_	609.2242	609.2252	1.6	20	609.2282(46.9); 472.1428(8.3); 446.1633(96.8); 431.1391(26.4); 283.0979(100); 282.0901(5.9); 268.0743(29.0)
**36**	12.721	*N*-caffeoyltyramine dimer (e.g., Cannabisin M)	C_34_H_32_N_2_O_8_	595.2086	595.2106	3.4	20	298.1088(100); 178.0505(2.6); 135.0457(5.4)
**37**	12.997	Cannabisin F	C_36_H_36_N_2_O_8_	623.2399	623.2416	2.7	20	623.2463(21.7); 486.1595(7.4); 460.1799(82.6); 445.1561(33.9); 430.1324(29.9); 297.1144(100); 296.1064(9.7); 282.0905(29.6); 267.0669(15.2)
**38**	13.251	*N*-caffeoyltyramine dimer (e.g., Cannabisin Q)	C_34_H_32_N_2_O_8_	595.2086	595.2094	1.4	20	298.1088(100); 178.0506(2.2); 135.0457(3.8)
**39**	14.178	Grossamide	C_36_H_36_N_2_O_8_	623.2399	623.2415 659.2180 [M + Cl]^−^	2.6	20	623.2432(19.8); 486.1579(7.4); 460.1778(7.2); 445.1545(43.5); 430.1306(32.8); 297.1129(100); 296.1049(11.1); 282.0892(35.7); 267.0656(16.6)

**Table 3:** TOF-MS and TOF-MS/MS of tentatively identified flavonol glycosides in the investigated hempseed fraction. RT = Retention Time; RDB = Ring Double Bond equivalent value. Compounds were numbered based on their RT in the whole LnHS chromatogram.

	RT (min)	Tentative Assignment	Formula	[M − H]^−^ calc. (*m/z*)	[M − H]^−^ found (*m/z*)	Error (ppm)	RDB	MS/MS Fragment Ions (*m/z*) and Relative Intensity (%)
**3**	6.204	Quercetin pentoside	C_20_H_18_O_11_	433.0776	433.0777	0.1	12	433.0823(9.8); 301.0362(29.4); 300.0273(100); 271.0250(69.4); 255.0304(31.7); 243.0304(12.2); 227.0358(5.3); 151.0036(10.3)
**7**	6.829	Quercetin-*O*-deoxyhexoside	C_21_H_20_O_11_	447.0933	447.0937	0.9	12	447.0947(8.1); 301.0357(59.7); 300.0276(100); 271.0244(56.9); 255.0294(36.2); 243.0291(12.1); 227.0344(7.2); 178.9988(6.6); 151.0036(14.1)
**9**	7.157	Kaempferol pentoside 1	C_20_H_18_O_10_	417.0827	417.0828	0.2	12	417.0837(16.3); 285.0406(14.1); 284.0328(80.4); 256.0378(8.1); 255.0299(100); 227.0352(77.8); 211.0398(5.4)
**10**	7.596	Kaempferol pentoside 2	C_20_H_18_O_10_	417.0827	417.0832	1.1	12	417.0848(7.1); 285.0408(34.4); 284.0329 (78.6); 256.0373(7.7); 255.0303 (100); 229.0503(8.9); 227.0351(71.4); 183.0452(5.3)
**12**	8.011	Kaempferol-*O*-deoxyhexoside 1	C_21_H_20_O_10_	431.0984	431.0984	0.1	12	431.1010(10.5); 285.0413(100); 284.0333(80.2); 257.0463(7.1); 256.0388(6.8); 255.0308(94.1); 229.0508(13.8); 227.0355(70.0); 211.0406(5.9); 183.0455(5.6)
**14**	8.032	Kaempferol-*O*-deoxyhexoside 2	C_12_H_22_O_6_	431.0984	431.0990	1.5	12	431.0988(12.6); 285.0400(82.9); 284.0321(92.7); 272.9218(3.3); 257.0443(5.6); 256.0360(5.9); 255.0294(100); 239.0351(3.2); 229.0501(11.3); 227.0346(61.7); 211.0399(5.4); 197.0590(3.4); 187.0385(5.3); 183.0452(3.4); 169.0649(1.7); 163.0035(2.4); 159.0452(1.6)
**18**	8.561	Quercetin-7-*O*-acetyldeoxyhexose	C_23_H_22_O_12_	489.1039	489.1040	0.3	13	489.1062(9.2); 429.0826(2.1); 409.1575(1.6); 397.1557(2.0); 315.0667(2.4); 301.0361(14.3); 300.0278(100); 299.0194(1.8); 271.0256(42.8); 269.0716(9.8); 255.0301(21.6); 243.0303(6.5); 227.0367(14.0); 178.9995(11.1); 151.0048(9.3)

## Supplementary Materials

The following are available online: Figure S1. Simplified extraction and fractionation scheme of cryo-crushed hempseeds. Figure S2. Morphological changes in SH-SY5Y cells treated with LnHS fraction in respect to untreated cells. Representative images were acquired by Inverted Phase Contrast Brightfield Zeiss Primo Vert Microscope. Figure S3. A) TOF-MS/MS spectrum; B) proposed fragmentation pathway of the [M − H]^−^ ion; C) UV-DAD spectrum for compound **2**. In B panel, the theoretical *m/z* value is reported below each structure. Figure S4. A) Extracted ion chromatogram (XIC) of the [M − H]^−^ ion at *m/z* 312.124 ±0.025; TOF-MS/MS spectra of B) compound 8, and C) compound **17**. In the grey panel the tentatively proposed fragmentation pathway of the [M − H]^−^ ions. Representative UV-DAD spectrum, acquired under peak **17**, is reported in panel D. Figure S5. A) TOF-MS spectrum for compound **11**; B) TOF MS/MS spectrum of the [M − H]^−^ ion; C) UV-DAD spectrum. Figure S6. A) TOF-MS/MS spectrum and B) proposed fragmentation pathway of the [M − H]^−^ ion for compound **33**; theoretical *m/z* values are reported below each structure. Figure S7. Compound 16 A) UV-DAD spectrum; B) TOF-MS/MS spectrum (in grey panel TOF-MS spectrum is reported. C) Proposed fragmentation pathway of the [M − H]^−^ ion (theoretical *m/z* values are reported below each structure). Figure S8. Proposed fragmentation pathway of the [M − H]^−^ ion relative to cannabisin B isomers (theoretical *m/z* values are reported below each structure). Figure S9. A) Proposed fragmentation pathway of the [M − H]^−^ ion of the compound **28** (theoretical *m/z* values are reported below each structure); B) UV-DAD, and C) TOF-MS/MS spectra. Figure S10. TOF-MS/MS spectra of compounds **32**, **36** and **38** (A, B, and C, respectively). Proposed fragmentation pathway of the [M − H]^−^ ion of the compound **32** is reported in grey panel, whereas that of **36** and **38** is in light green panel (theoretical *m/z* values are reported below each structure). Figure S11. TOF-MS/MS spectra of compounds **4** and **6** (A, and B, respectively). Proposed fragmentation pathway of the [M − H]^−^ ion of both the compounds are reported in grey panels (theoretical *m/z* values are reported below each structure). Figure S12. TOF-MS/MS spectrum (A) and proposed fragmentation pathway of the [M − H]^−^ ion of compound **13** (theoretical *m/z* values are reported below each structure). Figure S13. TOF-MS/MS and proposed fragmentation pathway of the [M − H]^−^ ion for compound **26**. The theoretical *m/z* value is reported below each structure. Figure S14. TOF-MS/MS spectra of compounds A) **27**, B) **29**, C) **37**, and D) **39**. Figure S15. A) TOF-MS/MS spectrum of compound **30**, and B) proposed fragmentation pathway of its [M − H]^−^ ion (theoretical *m/z* values are reported below each structure). Figure S16. Proposed fragmentation pathway of the [M − H]^−^ ion for compounds **31** and **34**. Figure S17. Flavanol glycosides in LnHS hempseed fraction. Figure S18. TOF-MS/MS spectra of quercetin derivatives A) **3**, B) **7** and C) **18**. Figure S19. A) Extracted ion chromatogram (XIC) of the [M − H]^−^ ion at *m/z* 417.083 ±0.025; TOF-MS/MS spectra of B) compound **9**, and C) compound **10**. Figure S20. TOF-MS/MS spectra of kaempferol derivatives A) **12**, and B) **14**. Figure S21. A) Relative content of each class of the tentatively identified compounds: HAAs – hydroxycinnamoyl amides; LnAs – lignanamides; Fls – Flavonols; B) relative content of lignanamides sharing a common [M − H]^−^ ion in LnHS fraction. Figure S22. A) mRNA expression of Sirt1 and Sirt2 by real-time; B) mRNA expression of IL6 and IL10 by real-time in U-87 and HF cells after 24 and 48 h exposure times.

## References

[1] Adefegha A. (2017). Functional Foods and Nutraceuticals as Dietary Intervention in Chronic Diseases; Novel Perspectives for Health Promotion and Disease Prevention. J. Diet. Suppl..

[2] Piccolella S., Crescente G., Candela L., Pacifico S. (2019). Nutraceutical polyphenols: New analytical challenges and opportunities. J. Pharm. Biomed. Anal..

[3] Piccolella S., Pacifico S., Fishbein J.C., Heilman J.M. (2015). Plant-Derived Polyphenols: A Chemopreventive and Chemoprotectant Worth-Exploring Resource in Toxicology. Advances in Molecular Toxicology.

[4] García C.R., Sánchez-Quesada C., Toledo E., Rodríguez-Delgado M., Gaforio J.J. (2019). Naturally Lignan-Rich Foods: A Dietary Tool for Health Promotion?. Molecules.

[5] Gaya P., Medina M., Sanchez A., Landete J.M. (2016). Phytoestrogen Metabolism by Adult Human Gut Microbiota. Molecules.

[6] Zálešák F., Bon D., Pospisil J. (2019). Lignans and Neolignans: Plant secondary metabolites as a reservoir of biologically active substances. Pharmacol. Res..

[7] Kajla P., Sharma A., Sood D.R. (2014). Flaxseed—a potential functional food source. J. Food Sci. Technol..

[8] Sicilia T., Niemeyer H.B., Honig D.M., Metzler M. (2003). Identification and Stereochemical Characterization of Lignans in Flaxseed and Pumpkin Seeds. J. Agric. Food Chem..

[9] André C.M., Hausman J.-F., Guerriero G. (2016). Cannabis sativa: The Plant of the Thousand and One Molecules. Front. Plant Sci..

[10] Crescente G., Piccolella S., Esposito A., Scognamiglio M., Fiorentino A., Pacifico S. (2018). Chemical composition and nutraceutical properties of hempseed: an ancient food with actual functional value. Phytochem. Rev..

[11] Chen T., Hao J., He J., Zhang J., Li Y., Liu R., Li L. (2013). Cannabisin B induces autophagic cell death by inhibiting the AKT/mTOR pathway and S phase cell cycle arrest in HepG2 cells. Food Chem..

[12] Moccia S., Siano F., Russo G.L., Volpe M.G., La Cara F., Pacifico S., Piccolella S., Picariello G. (2019). Antiproliferative and antioxidant effect of polar hemp extracts (Cannabis sativa L., Fedora cv.) in human colorectal cell lines. Int. J. Food Sci. Nutr..

[13] Gallagher R.S., Ananth R., Granger K., Bradley B., Anderson J., Fuerst E.P. (2010). Phenolic and Short-Chained Aliphatic Organic Acid Constituents of Wild Oat (*Avena fatua* L.) Seeds. J. Agric. Food Chem..

[14] Zhang B., Huang R., Hua J., Liang H., Pan Y., Dai L., Liang D., Wang H.-S. (2016). Antitumor lignanamides from the aerial parts of Corydalis saxicola. Phytomedicine.

[15] Li C.-X., Song X.-Y., Zhao W.-Y., Yao G.-D., Lin B., Huang X.-X., Li L.-Z., Song S.-J. (2019). Characterization of enantiomeric lignanamides from *Solanum nigrum* L. and their neuroprotective effects against MPP+-induced SH-SY5Y cells injury. Phytochemistry.

[16] Sun J., Gu Y.-F., Su X.-Q., Li M.-M., Huo H.-X., Zhang J., Zeng K.-W., Zhang Q., Zhao Y.-F., Li J. (2014). Anti-inflammatory lignanamides from the roots of Solanum melongena L. Fitoterapia.

[17] Zhang J.-X., Guan S.-H., Feng R.-H., Wang Y., Wu Z.-Y., Zhang Y.-B., Chen X.-H., Bi K.-S., Guo D.-A. (2013). Neolignanamides, Lignanamides, and Other Phenolic Compounds from the Root Bark of *Lycium chinense*. J. Nat. Prod..

[18] Zhang J., Guan S., Sun J., Liu T., Chen P., Feng R., Chen X., Wu W.-Y., Yang M., Guo D.-A. (2014). Characterization and profiling of phenolic amides from Cortex Lycii by ultra-high performance liquid chromatography coupled with LTQ-Orbitrap mass spectrometry. Anal. Bioanal. Chem..

[19] Zhu G.-Y., Yang J., Yao X., Yang X., Fu J., Liu X., Bai L.-P., Liu L., Jiang Z.-H. (2018). (±)-Sativamides A and B, Two Pairs of Racemic Nor-Lignanamide Enantiomers from the Fruits of Cannabis sativa. J. Org. Chem..

[20] Yan X., Tang J., Passos C.D.S., Nurisso A., Simoes-Pires C., Ji M., Lou H., Fan P. (2015). Characterization of Lignanamides from Hemp (*Cannabis sativa* L.) Seed and Their Antioxidant and Acetylcholinesterase Inhibitory Activities. J. Agric. Food Chem..

[21] Wang S., Luo Q., Fan P. (2019). Cannabisin F from Hemp (*Cannabis sativa*) Seed Suppresses Lipopolysaccharide-Induced Inflammatory Responses in BV2 Microglia as SIRT1 Modulator. Int. J. Mol. Sci..

[22] Diao W., Tong X., Yang C., Zhang F., Bao C., Chen H., Liu L., Li M., Ye F., Fan Q. (2019). Behaviors of Glioblastoma Cells in in Vitro Microenvironments. Sci. Rep..

[23] Louis D.N., Perry A., Reifenberger G., Deimling A., Figarella-Branger M., Cavenee W.K., Ohgaki H., Wiestler O.D., Kleihues P., Ellison D.W. (2016). The 2016 World Health Organization Classification of Tumors of the Central Nervous System: a summary. Acta Neuropathol..

[24] Faugno S., Piccolella S., Sannino M., Principio L., Crescente G., Baldi G.M., Fiorentino N., Pacifico S. (2019). Can agronomic practices and cold-pressing extraction parameters affect phenols and polyphenols content in hempseed oils?. Ind. Crop. Prod..

[25] Smeriglio A., Galati E.M., Monforte M.T., Lanuzza F., D’Angelo V., Circosta C. (2016). Polyphenolic Compounds and Antioxidant Activity of Cold-Pressed Seed Oil from Finola Cultivar of *Cannabis sativa* L. Phytotherapy Res..

[26] Zhou Y., Wang S., Lou H., Fan P. (2018). Chemical constituents of hemp (*Cannabis sativa* L.) seed with potential anti-neuroinflammatory activity. Phytochem. Lett..

[27] Bourjot M., Zedet A., Demange B., Pudlo M., Girard-Thernier C. (2017). In Vitro Mammalian Arginase Inhibitory and Antioxidant Effects of Amide Derivatives Isolated from the Hempseed Cakes (*Cannabis sativa*). Planta Medica Int. Open.

[28] Simón-Manso Y., Neta P., Yang X., Stein S.E. (2011). Loss of 45 Da from a2 Ions and Preferential Loss of 48 Da from a2 Ions Containing Methionine in Peptide Ion Tandem Mass Spectra. J. Am. Soc. Mass Spectrom..

[29] Fokoue H.H., Marques J.V., Correia M.V., Yamaguchi L.F., Qu X., Aires-De-Sousa J., Scotti M.T., Lopes N.P., Kato M. (2018). Fragmentation pattern of amides by EI and HRESI: study of protonation sites using DFT-3LYP data. RSC Adv..

[30] Matsuda F., Miyagawa H., Ueno T. (2000). Beta-1,3-glucooligosaccharide induced activation of four enzymes responsible for N-p-coumaroyloctopamine biosynthesis in potato (*Solanum tuberosum* cv.) tuber tissue. Z. für Nat. C.

[31] Ko H.-J., Ahn E.-K., Oh J.S. (2015). N-trans-*p*-caffeoyl tyramine isolated from *Tribulus terrestris* exerts anti-inflammatory effects in lipopolysaccharide-stimulated RAW 264.7 cells. Int. J. Mol. Med..

[32] Takayama M. (2001). N-Cα bond cleavage of the peptide backbone via hydrogen abstraction. J. Am. Soc. Mass Spectrom..

[33] Chen C.-Y., Yeh Y.-T., Yang W.-L. (2011). Amides from the stem of *Capsicum annuum*. Nat. Prod. Commun..

[34] Wang S., Suh J.H., Hung W.-L., Zheng X., Wang Y., Ho C.-T. (2018). Use of UHPLC-TripleQ with synthetic standards to profile anti-inflammatory hydroxycinnamic acid amides in root barks and leaves of *Lycium barbarum*. J. Food Drug Anal..

[35] Santos L., Boaventura M., De Oliveira A., Cassady J. (1996). Grossamide and *N*-*trans*-caffeoyltyramine from *Annona crassiflora* Seeds. Planta Medica.

[36] Neelam S., Gokara M., Sudhamalla B., Amooru D.G., Subramanyam R. (2010). Interaction studies of coumaroyltyramine with human serum albumin and its biological importance. J. Phys. Chem. B.

[37] Walters D., Meurer–Grimes B., Rovira I. (2001). Antifungal activity of three spermidine conjugates. FEMS Microbiol. Lett..

[38] Gericke S., Lübken T., Wolf D., Kaiser M., Hannig C., Speer K. (2018). Identification of new compounds from sage flowers (*Salvia officinalis* L.) as markers for quality control and the influence of the manufacturing technology on the chemical composition and antibacterial activity of sage flower extracts. J. Agric. Food Chem..

[39] Sakakibara I., Katsuhara T., Ikeya Y., Hayashi K., Mitsuhashi H. (1991). Cannabisin A, an arylnaphthalene lignanamide from fruits of *Cannabis sativa*. Phytochemistry.

[40] Hao G., Wang D., Gu J., Shen Q., Gross S.S., Wang Y. (2009). Neutral loss of isocyanic acid in peptide CID spectra: A novel diagnostic marker for mass spectrometric identification of protein citrullination. J. Am. Soc. Mass Spectrom..

[41] Morreel K., Dima O., Kim H., Lu F., Niculaes C., Vanholme R., Dauwe R., Goeminne G., Inze D., Messens E. (2010). Mass spectrometry-based sequencing of lignin oligomers. Plant Physiol..

[42] Seca A.M.L., Silva A.M.S., Silvestre A.J.D., Cavaleiro J.A.S., Domingues F.M., Neto C. (2001). Lignanamides and other phenolic constituents from the bark of kenaf (*Hibiscus cannabinus*). Phytochemistry.

[43] Bolleddula J., Fitch W., Vareed S.K., Nair M. (2012). Identification of metabolites in *Withania sominfera* fruits by liquid chromatography and high-resolution mass spectrometry. Rapid Commun. Mass Spectrom..

[44] Luo Q., Yan X., Bobrovskaya L., Ji M., Yuan H., Lou H., Fan P. (2017). Anti-neuroinflammatory effects of grossamide from hemp seed via suppression of TLR-4-mediated NF-κB signaling pathways in lipopolysaccharide-stimulated BV2 microglia cells. Mol. Cell. Biochem..

[45] Kim K.H., Moon E., Kim S.Y., Lee K.R. (2010). Lignans from the tuber-barks of *Colocasia antiquorum* var. *esculenta* and their antimelanogenic activity. J. Agric. Food Chem..

[46] Davis B.D., Brodbelt J. (2008). An investigation of the homolytic saccharide cleavage of deprotonated flavonol 3-O-glycosides in a quadrupole ion trap mass spectrometer. J. Mass Spectrom..

[47] Pacifico S., Piccolella S., Nocera P., Tranquillo E., Poggetto G.D., Catauro M. (2019). New insights into phenol and polyphenol composition of *Stevia rebaudiana* leaves. J. Pharm. Biomed. Anal..

[48] Jalili-Nik M., Sabri H., Zamiri E., Soukhtanloo M., Roshan M.K., Hosseini A., Mollazadeh H., Vahedi M.M., Afshari A.R., Mousavi S.H. (2019). Cytotoxic effects of *Ferula latisecta* on human glioma U87 Cells. Drug Res..

[49] Chan F.K., Moriwaki K., De Rosa M.J. (2013). Detection of necrosis by release of lactate dehydrogenase activity. Breast Cancer.

[50] Franken N.A., Rodermond H.M., Stap J., Haveman J., Van Bree C. (2006). Clonogenic assay of cells in vitro. Nat. Protoc..

[51] Gu J.-J., Gao G.-Z., Zhang S.-M. (2015). miR-218 inhibits the migration and invasion of glioma U87 cells through the Slit2-Robo1 pathway. Oncol. Lett..

[52] Russell R.C., Tian Y., Yuan H., Park H.W., Chang Y.-Y., Kim J., Kim H., Neufeld T.P., Dillin A., Guan K.-L. (2013). ULK1 induces autophagy by phosphorylating Beclin-1 and activating VPS34 lipid kinase. Nature.

[53] Menon M.B., Dhamija S. (2018). Beclin 1 Phosphorylation - at the Center of Autophagy Regulation. Front. Cell Dev. Boil..

[54] Ricci A., Fiorentino A., Piccolella S., Golino A., Pepi F., D’Abrosca B., Letizia M., Monaco P. (2008). Furofuranic glycosylated lignans: a gas-phase ion chemistry investigation by tandem mass spectrometry. Rapid Commun. Mass Spectrom..

[55] Nigro E., Colavita I., Sarnataro D., Scudiero O., Zambrano G., Granata V., Daniele A., Carotenuto A., Galdiero S., Folliero V. (2015). An ancestral host defence peptide within human β-defensin 3 recapitulates the antibacterial and antiviral activity of the full-length molecule. Sci. Rep..

[56] Pacifico S., Gallicchio M., Lorenz P., Duckstein S.M., Potenza N., Galasso S., Marciano S., Fiorentino A., Stintzing F.C., Monaco P. (2014). Neuroprotective potential of *Laurus nobilis* antioxidant polyphenol-enriched leaf extracts. Chem. Res. Toxicol..

[57] Hynds R.E., Gowers K.H.C., Nigro E., Butler C.R., Bonfanti P., Giangreco A., Prele C.M., Janes S.M. (2018). Cross-talk between human airway epithelial cells and 3T3-J2 feeder cells involves partial activation of human MET by murine HGF. PLoS ONE.

[58] Nigro E., Schettino P., Polito R., Scudiero O., Monaco M.L., De Palma G.D., Daniele A. (2018). Adiponectin and colon cancer: evidence for inhibitory effects on viability and migration of human colorectal cell lines. Mol. Cell. Biochem..

[59] Benelli G., Pavela R., Petrelli R., Cappellacci L., Santini G., Fiorini D., Sut S., Zengin G., Canale A., Maggi F. (2018). The essential oil from industrial hemp (*Cannabis sativa* L.) by-products as an effective tool for insect pest management in organic crops. Ind. Crop. Prod..

[60] Fiorini D., Molle A., Nabissi M., Santini G., Benelli G., Maggi F. (2019). Valorizing industrial hemp (*Cannabis sativa* L.) by-products: cannabidiol enrichment in the inflorescence essential oil optimizing sample pre-treatment prior to distillation. Ind. Crop. Prod..

[61] Figueira I., Garcia G., Pimpão R.C., Terrasso A., Costa I., Almeida A.F., Tavares L., Pais T.F., Pinto P., Ventura M.R. (2017). Polyphenols journey through blood-brain barrier towards neuronal protection. Sci. Rep..

[62] Youdim K.A., Dobbie M.S., Kuhnle G., Proteggente A.R., Abbott N.J., Rice-Evans C. (2003). Interaction between flavonoids and the blood-brain barrier: in vitro studies. J. Neurochem..

[63] Pacifico S., Piccolella S., Marciano S., Galasso S., Nocera P., Piscopo V., Fiorentino A., Monaco P. (2014). LC-MS/MS profiling of a mastic leaf phenol enriched extract and its effects on H_2_O_2_ and Aβ (25–35) oxidative injury in SK-B-NE (C)-2 cells. J. Agric. Food Chem..

[64] Turunen B.J., Ge H., Oyetunji J., Desino K.E., Vasandani V., Güthe S., Himes R.H., Audus K.L., Seelig A., Georg G.I. (2008). Paclitaxel succinate analogs: anionic and amide introduction as a strategy to impart blood–brain barrier permeability. Bioorganic Med. Chem. Lett..

[65] Price T.O., Uras F., Banks W.A., Ercal N. (2006). A novel antioxidant *N*-acetylcysteine amide prevents gp120- and Tat-induced oxidative stress in brain endothelial cells. Exp. Neurol..

[66] Könczöl Á., Rendes K., Dékány M., Müller J., Riethmüller E., Balogh G.T. (2016). Blood-brain barrier specific permeability assay reveals N -methylated tyramine derivatives in standardised leaf extracts and herbal products of *Ginkgo biloba*. J. Pharm. Biomed. Anal..

