# Supplementary material for: Hempseed Lignanamides Rich-Fraction: Chemical Investigation and Cytotoxicity towards U-87 Glioblastoma Cells
Source: Molecules. 2020 Feb 26;25(5):1049. doi: 10.3390/molecules25051049 (PMC7179246; doi:10.3390/molecules25051049)
Supplement: Supplementary file 1 [file molecules-25-01049-s001.pdf]

molecules

Supplementary Information

Hempseed Lignanamides Rich-Fraction: Chemical Investigation and Cytotoxicity towards U-87 Glioblastoma Cells

Ersilia Nigro [1,2,†], Giuseppina Crescente [1,†], Marialuisa Formato [1,†], Maria Tommasina Pecoraro [1], Marta Mallardo [1,2], Simona Piccolella [1], Aurora Daniele [1,2] and Severina Pacifico [1,*]

[1] Department of Environmental Biological and Pharmaceutical Sciences and Technologies, University of Campania "Luigi Vanvitelli", Via Vivaldi 43, I-81100 Caserta, Italy; nigro@ceinge.unina.it (E.N.); giuseppina.crescente@unicampania.it (G.C.); marialuisa.formato@unicampania.it (M.F.); mariatommasina.pecoraro@unicampania.it (M.T.P.); marta.mallardo@unicampania.it (M.M.); simona.piccolella@unicampania.it (S.P.); aurora.daniele@unicampania.it (A.D.)

[2] CEINGE-Advanced Biotechnologies, Scarl, 80131 Napoli, Italy

† These authors equally contributed to the work

* Correspondence: severina.pacifico@unicampania.it; Tel.: +39-0823-274578

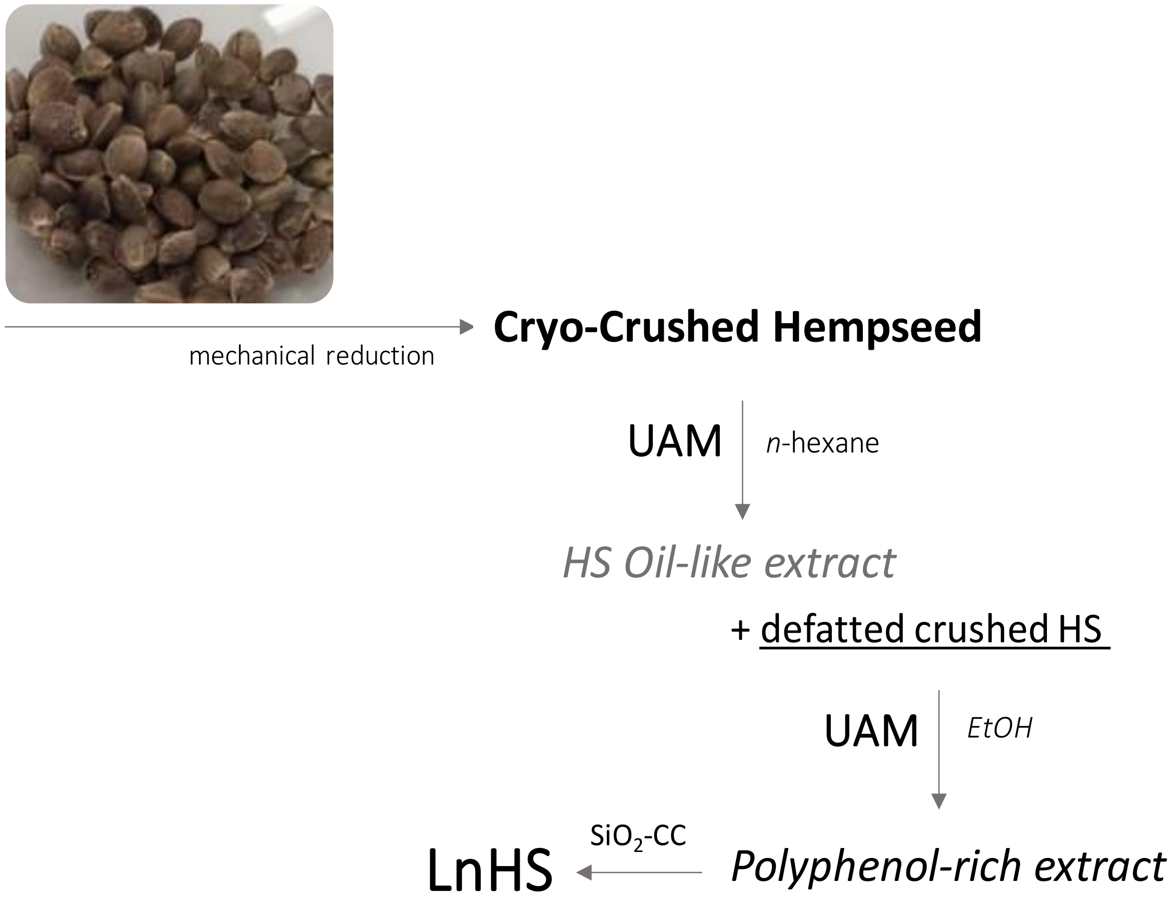

Figure 1. Simplified extraction and fractionation scheme of cryo-crushed hempseeds.

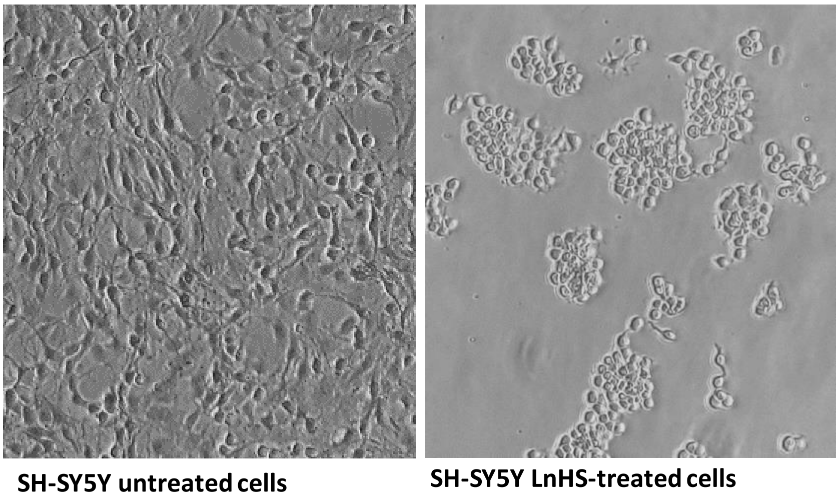

Figure 2. Morphological changes in SH-SY5Y cells treated with LnHS fraction in respect to untreated cells. Representative images were acquired by Inverted Phase Contrast Brightfield Zeiss Primo Vert Microscope.

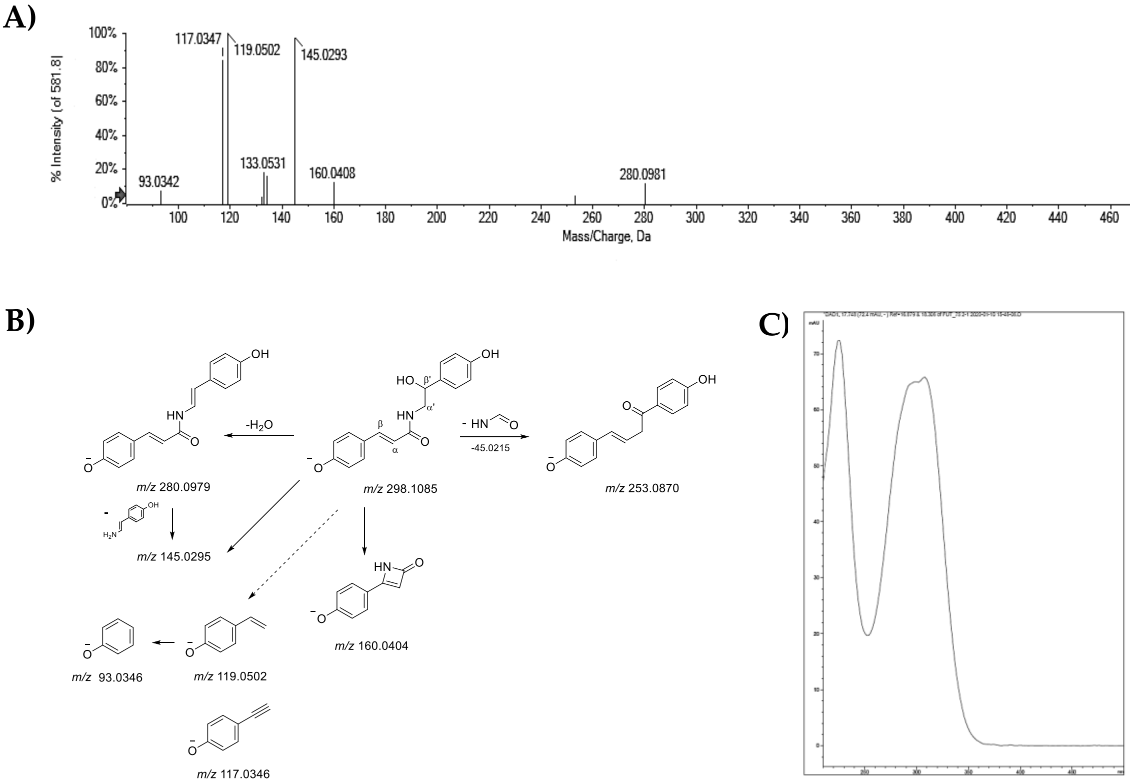

Figure 3. A) TOF-MS/MS spectrum; B) proposed fragmentation pathway of the [M-H]- ion; C) UV-DAD spectrum for compound **2**. In B panel, the theoretical *m*/*z* value is reported below each structure.

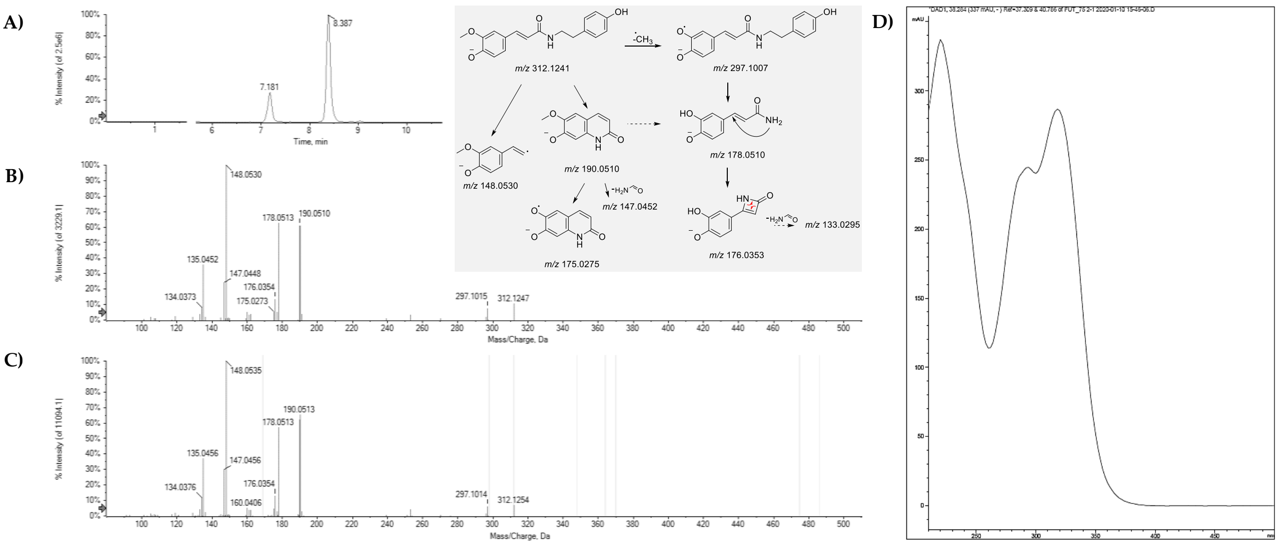

Figure 4. A) Extracted ion chromatogram (XIC) of the [M-H]$^-$ ion at *m/z* 312.124 ±0.025; TOF-MS/MS spectra of B) compound **8**, and C) compound **17**. In the grey panel the tentatively proposed fragmentation pathway of the [M-H]$^-$ ions. Representative UV-DAD spectrum, acquired under peak **17**, is reported in panel D.

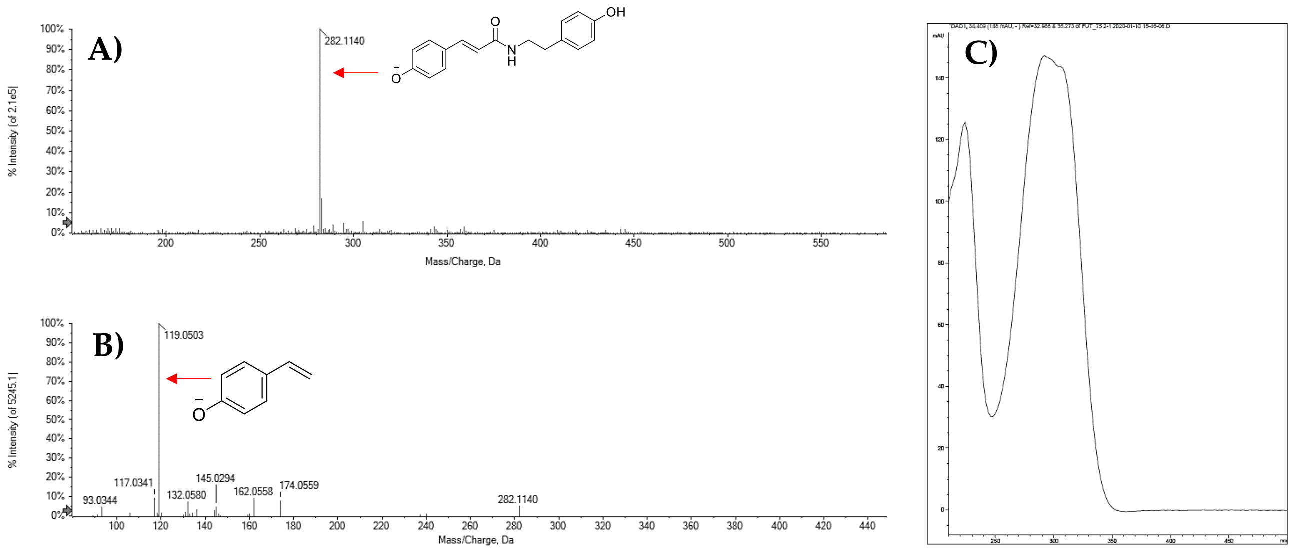

Figure 5. A) TOF-MS spectrum for compound **11**; B) TOF MS/MS spectrum of the [M-H]$^-$ ion**;** C) UV-DAD spectrum.

A)

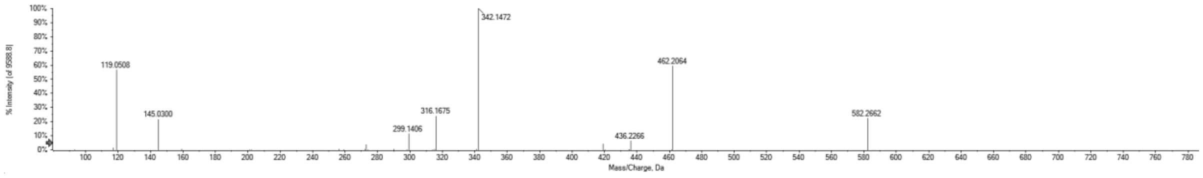

B)

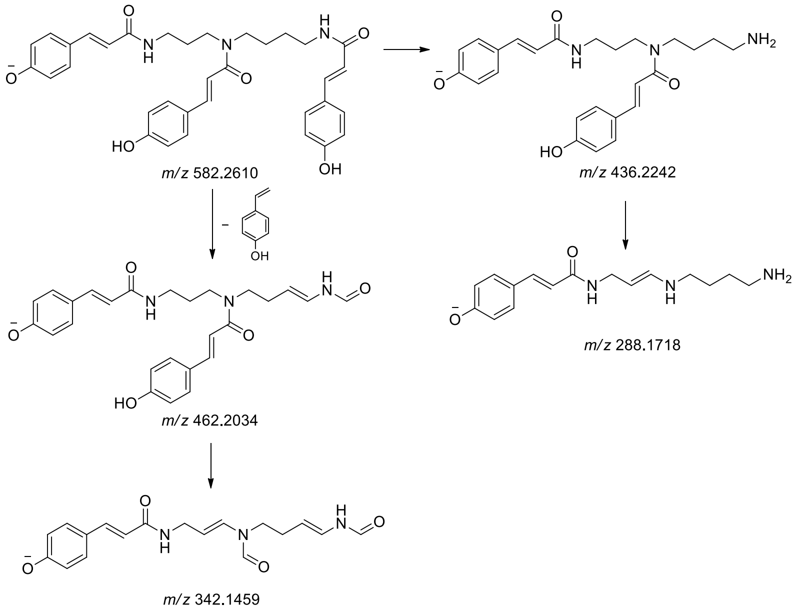

Figure 6. A) TOF-MS/MS spectrum and B) proposed fragmentation pathway of the [M-H]$^-$ ion for compound **33**; theoretical *m*/*z* values are reported below each structure.

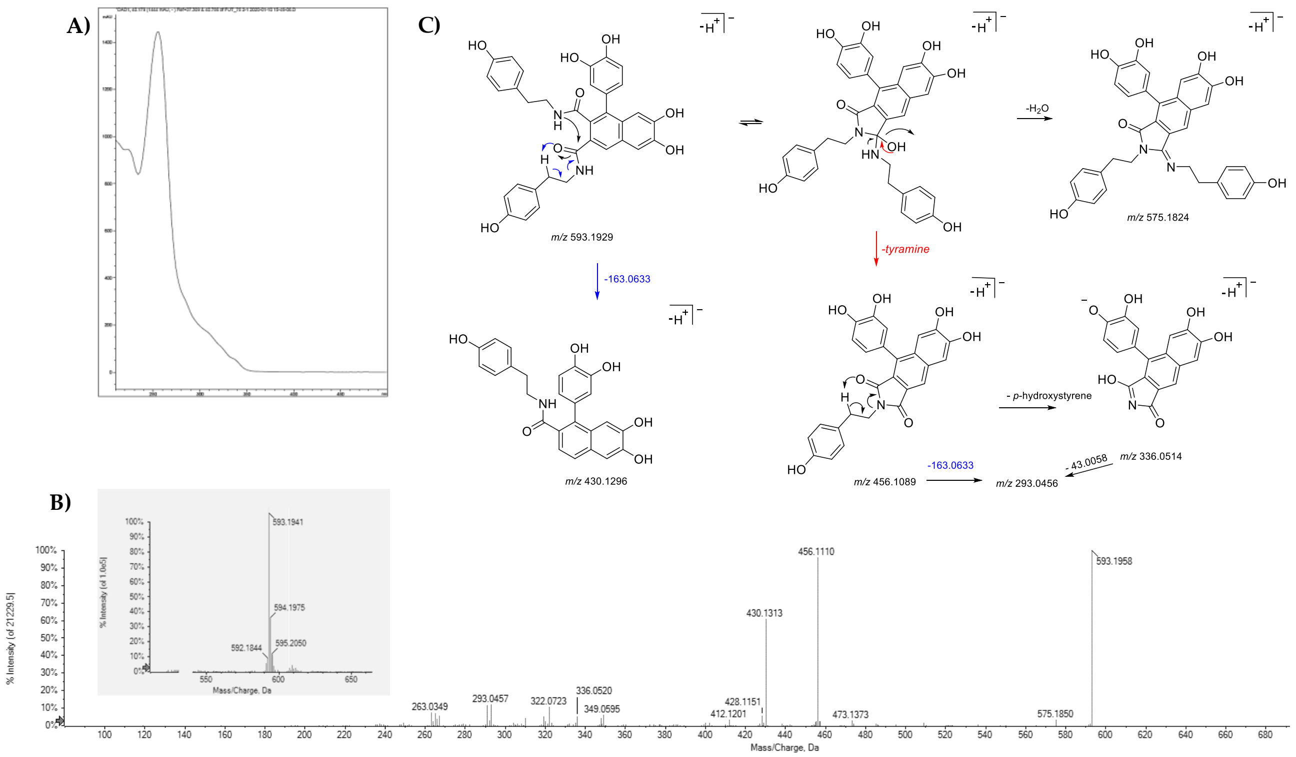

Figure 7. Compound **16** A) UV-DAD spectrum; B) TOF-MS/MS spectrum (in grey panel TOF-MS spectrum is reported. C) Proposed fragmentation pathway of the [M-H]- ion (theoretical *m*/*z* values are reported below each structure).

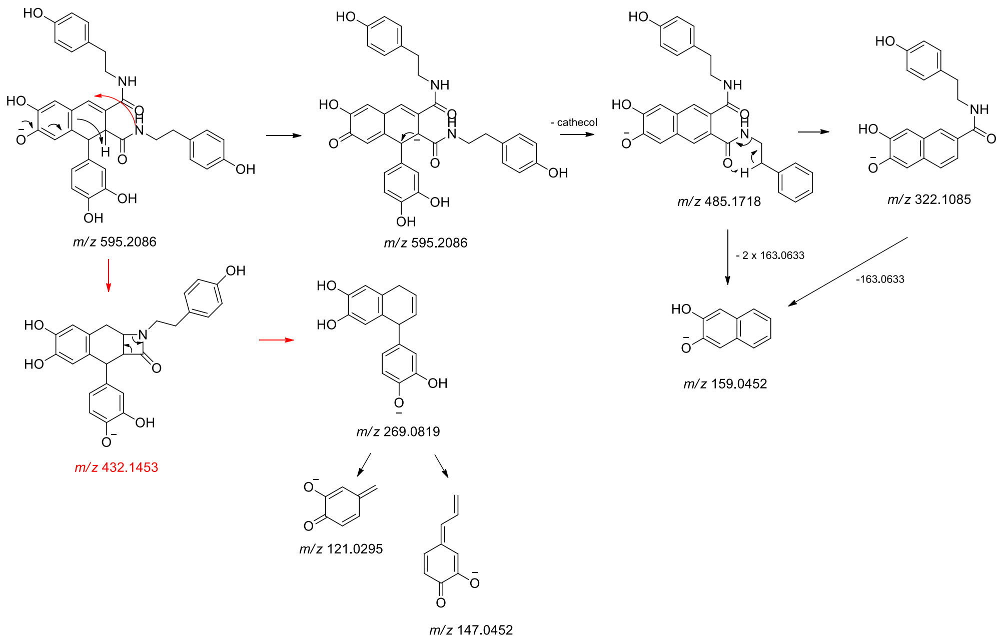

Figure 8. Proposed fragmentation pathway of the [M-H]- ion relative to cannabisin B isomers (theoretical *m/z* values are reported below each structure).

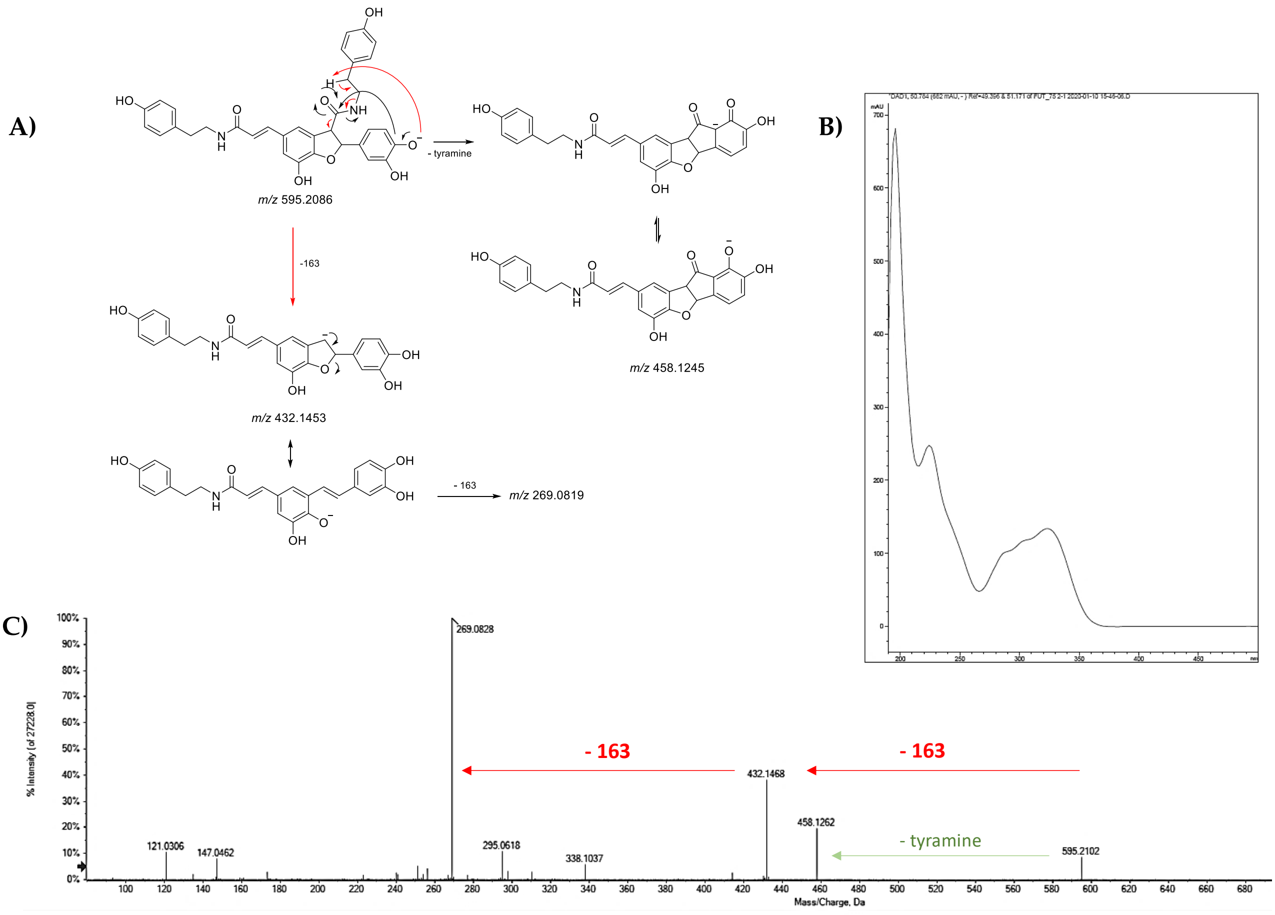

Figure 9. A) Proposed fragmentation pathway of the [M-H]$^-$ ion of the compound **28** (theoretical *m/z* values are reported below each structure); B) UV-DAD, and C) TOF-MS/MS spectra.

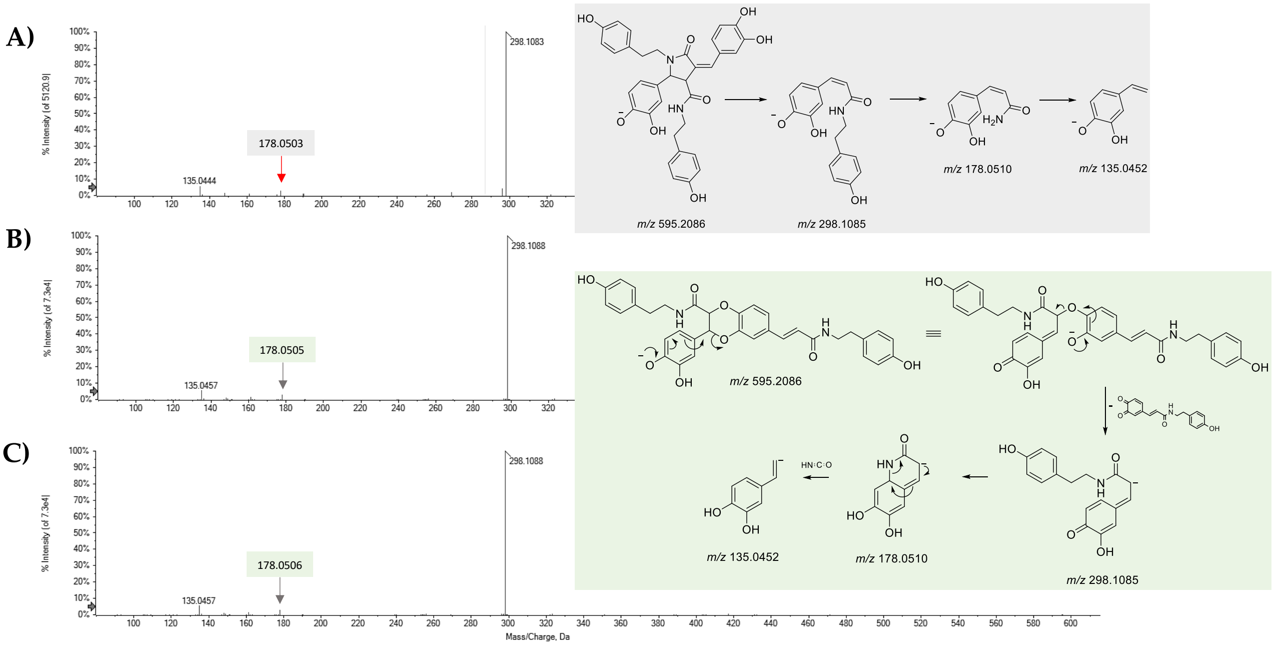

Figure 10. TOF-MS/MS spectra of compounds **32**, **36** and **38** (A, B, and C, respectively). Proposed fragmentation pathway of the $[M-H]^-$ ion of the compound **32** is reported in grey panel, whereas that of **36** and **38** is in light green panel (theoretical *m/z* values are reported below each structure).

A)

B)

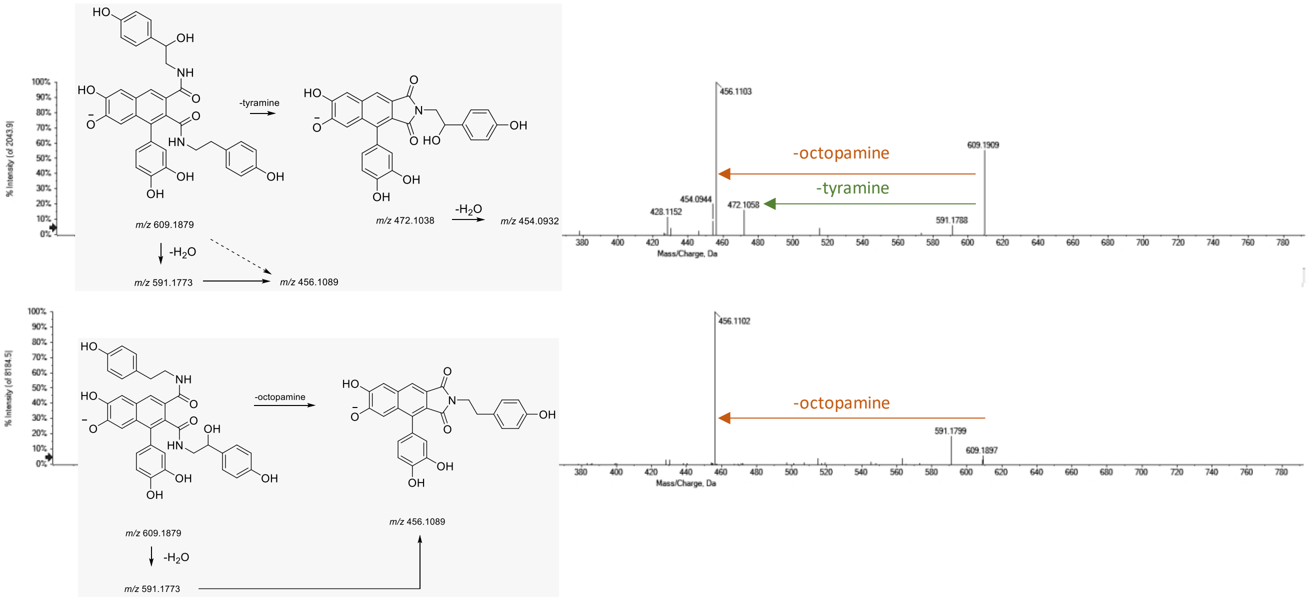

Figure 11. TOF-MS/MS spectra of compounds **4** and **6** (A, and B, respectively). Proposed fragmentation pathway of the [M-H]$^-$ ion of both the compounds are reported in grey panels (theoretical *m*/*z* values are reported below each structure).

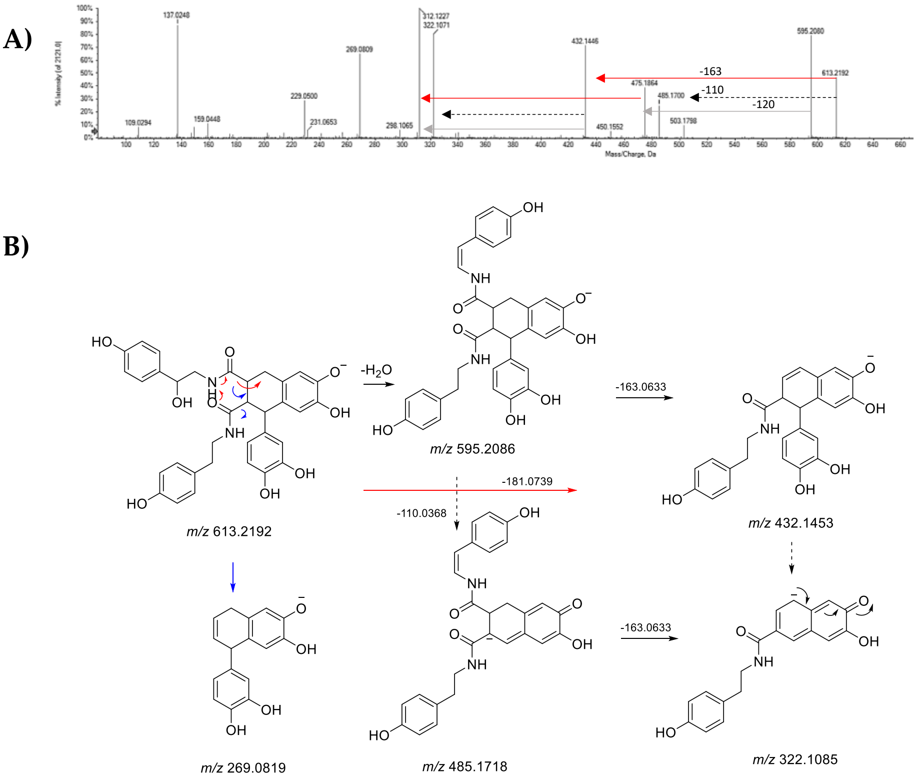

Figure S12. TOF-MS/MS spectrum (A) and proposed fragmentation pathway of the [M-H]- ion of compound **13** (theoretical *m*/*z* values are reported below each structure).

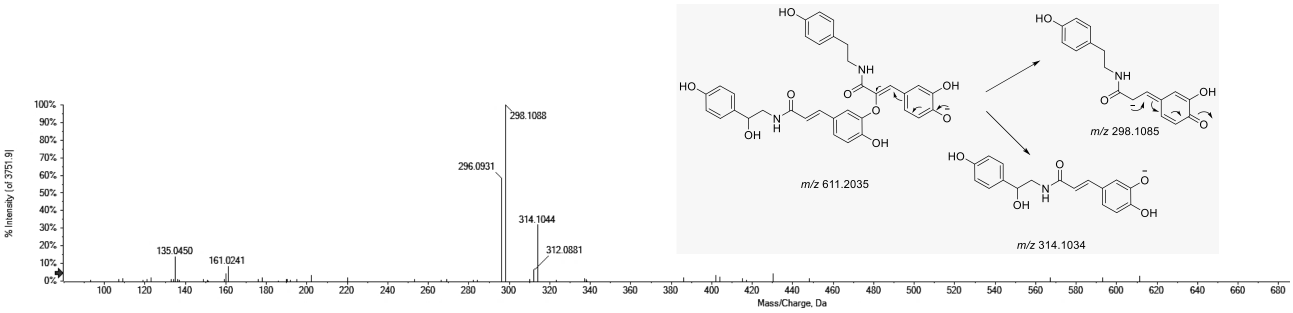

Figure S13. TOF-MS/MS and proposed fragmentation pathway of the [M-H]- ion for compound **26.** The theoretical *m*/*z* value is reported below each structure.

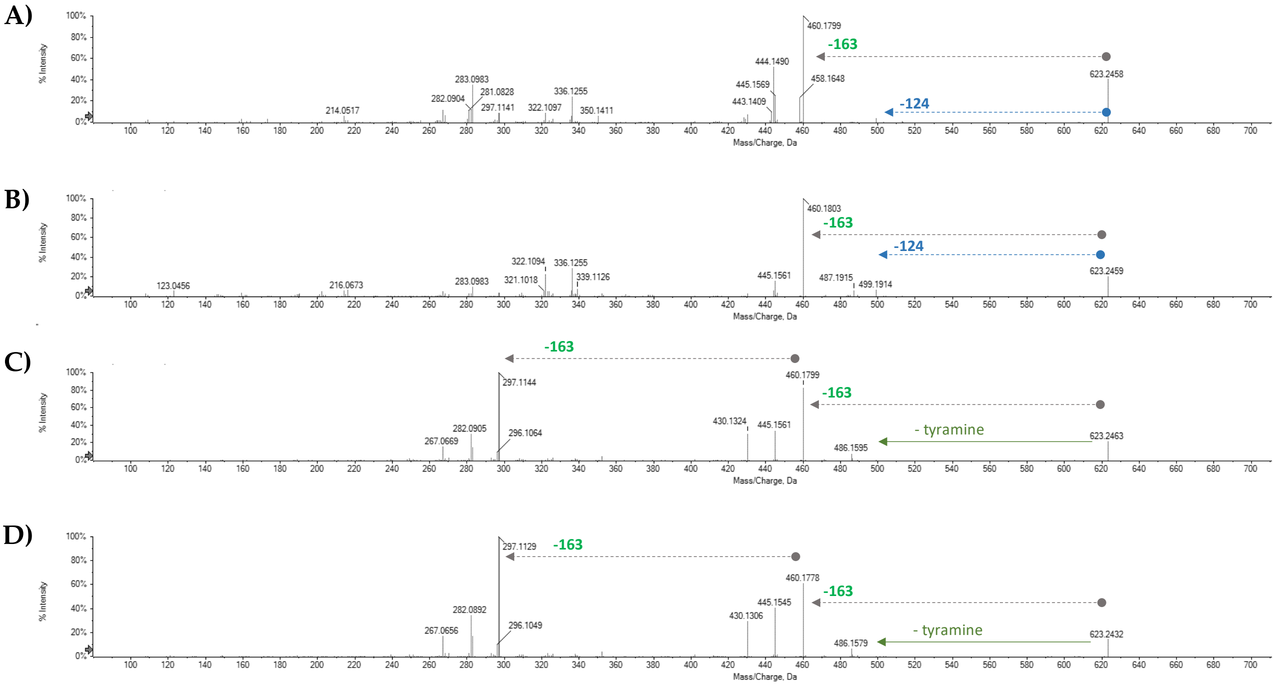

Figure 14. TOF-MS/MS spectra of compounds A) **27**, B) **29**, C) **37**, and D) **39**.

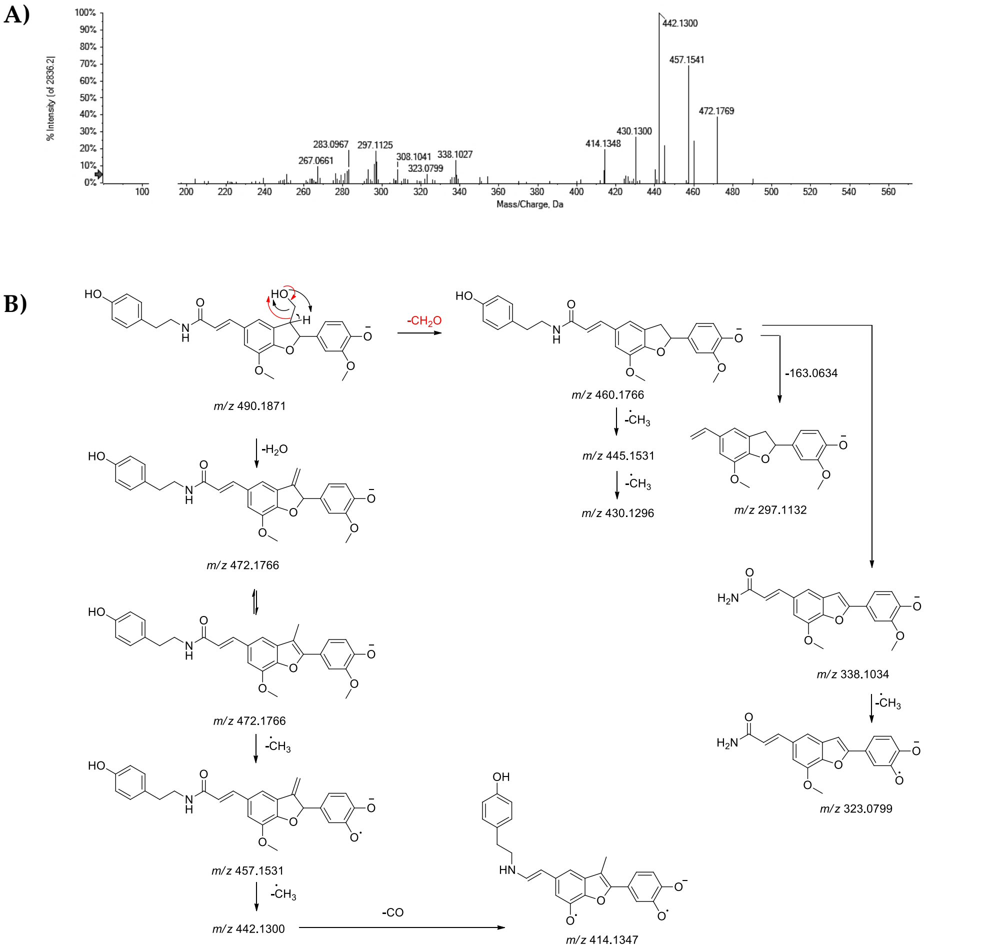

Figure 15. A) TOF-MS/MS spectrum of compound **30**, and B) proposed fragmentation pathway of its [M-H]⁻ ion (theoretical *m/z* values are reported below each structure).

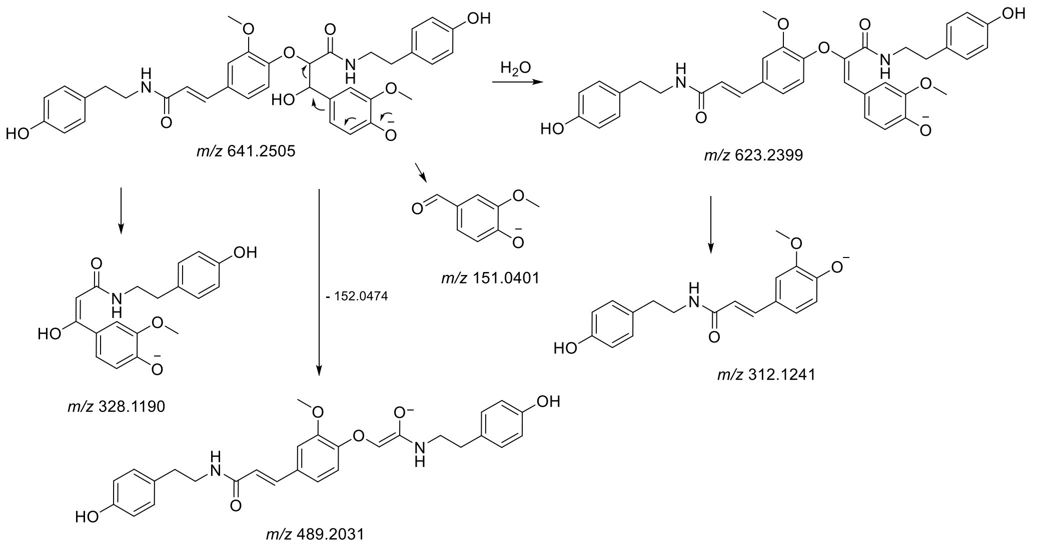

Figure 16. Proposed fragmentation pathway of the [M-H]- ion for compounds **31** and **34**.

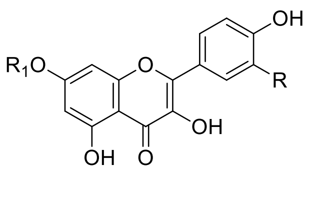

3 R = OH; R_1 = pentose
7 R = OH; R_1= deoxyhexose
9 R = H; R_1 = pentose
10 R = H; R_1 = pentose
12 R = H; R_1= deoxyhexose
14 R = H; R_1= deoxyhexose
18 R = OH; R_1= acetyldeoxyhexose

Figure 17. Flavanol glycosides in LnHS hempseed fraction.

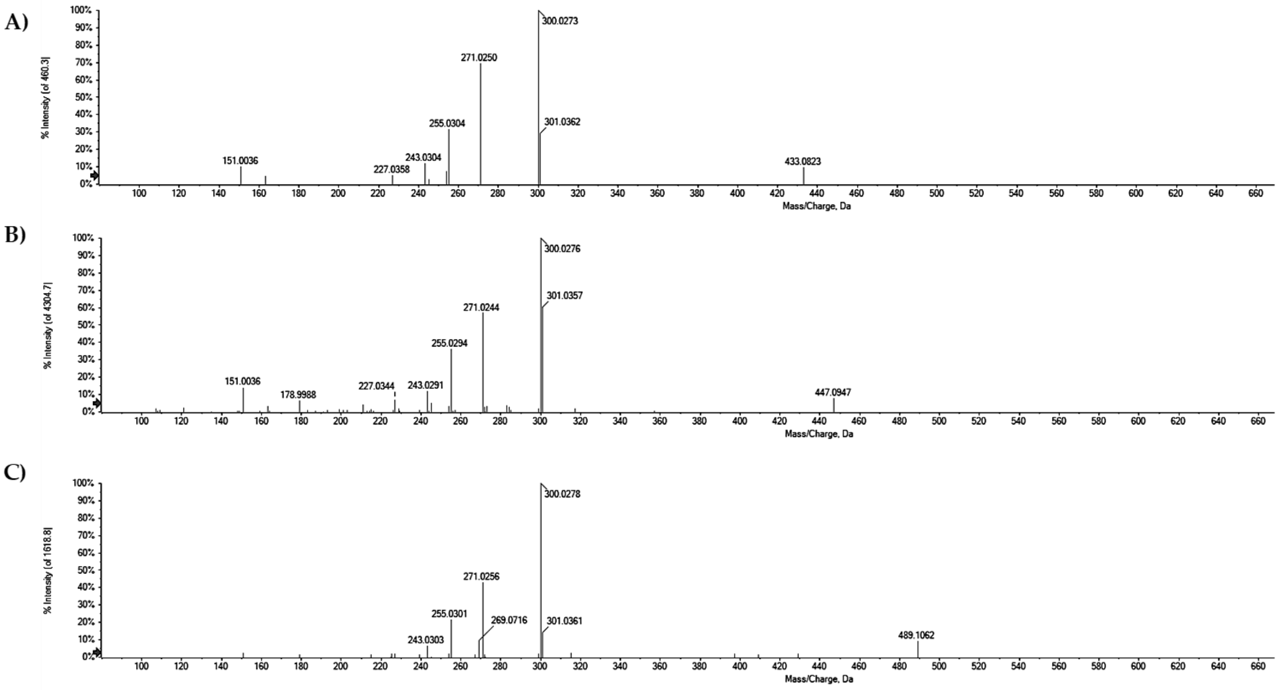

Figure 18. TOF-MS/MS spectra of quercetin derivatives A) **3**, B) **7** and C) **18**.

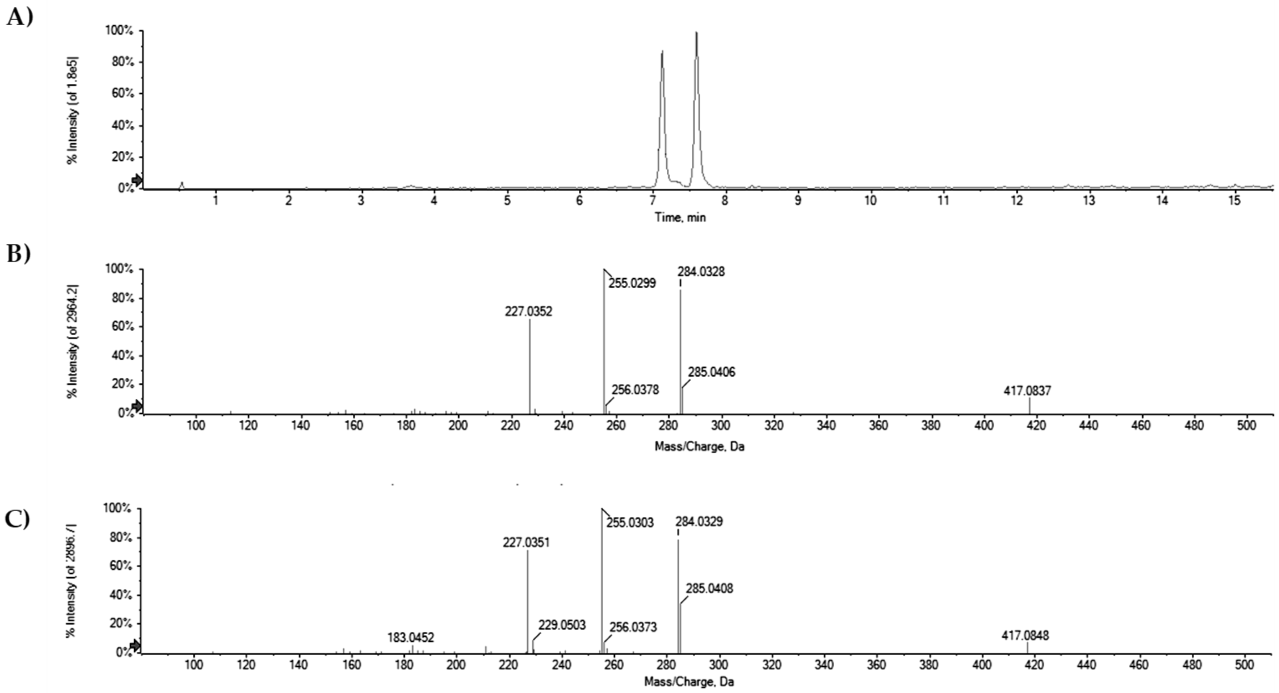

Figure 19. A) Extracted ion chromatogram (XIC) of the [M-H]$^-$ ion at *m*/*z* 417.083 ±0.025; TOF-MS/MS spectra of B) compound **9**, and C) compound **10**.

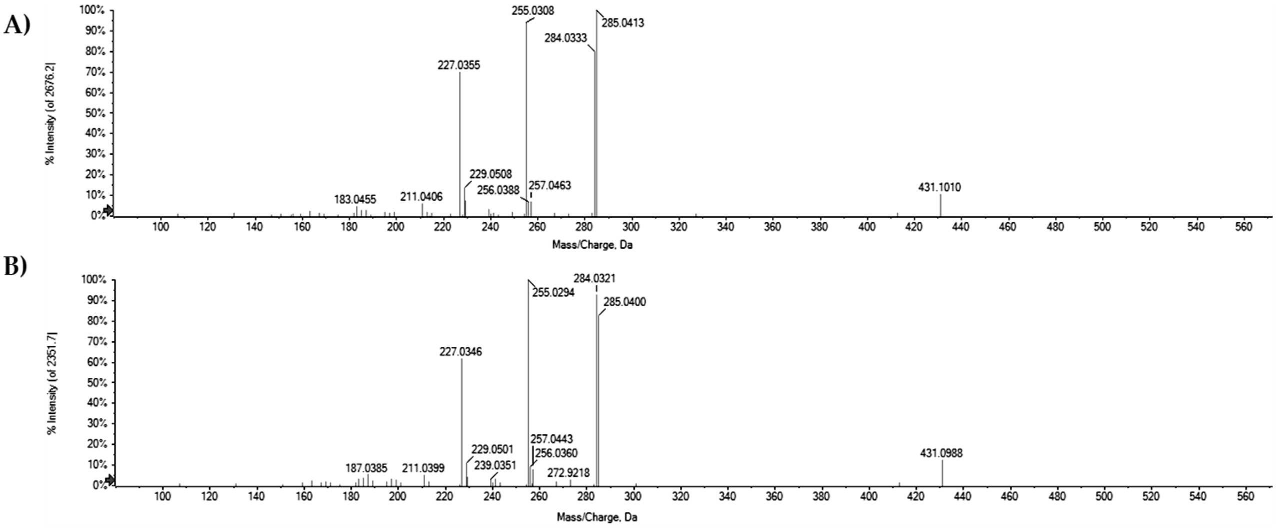

Figure 20. TOF-MS/MS spectra of kaempferol derivatives A) **12**, and B) **14**.

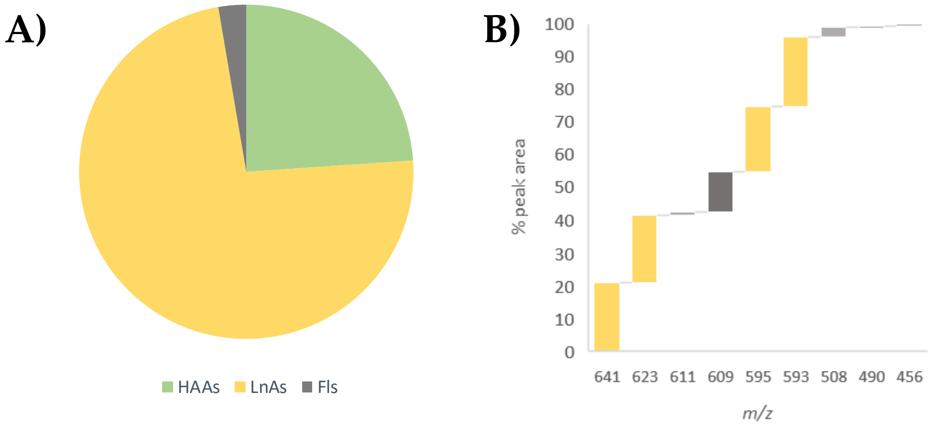

Figure 21. A) Relative content of each class of the tentatively identified compounds: HAAs – hydroxycinnamoyl amides; LnAs – lignanamides; Fls – Flavonols; B) Relative content of lignanamides sharing a common $[M-H]^-$ ion in LnHS fraction.

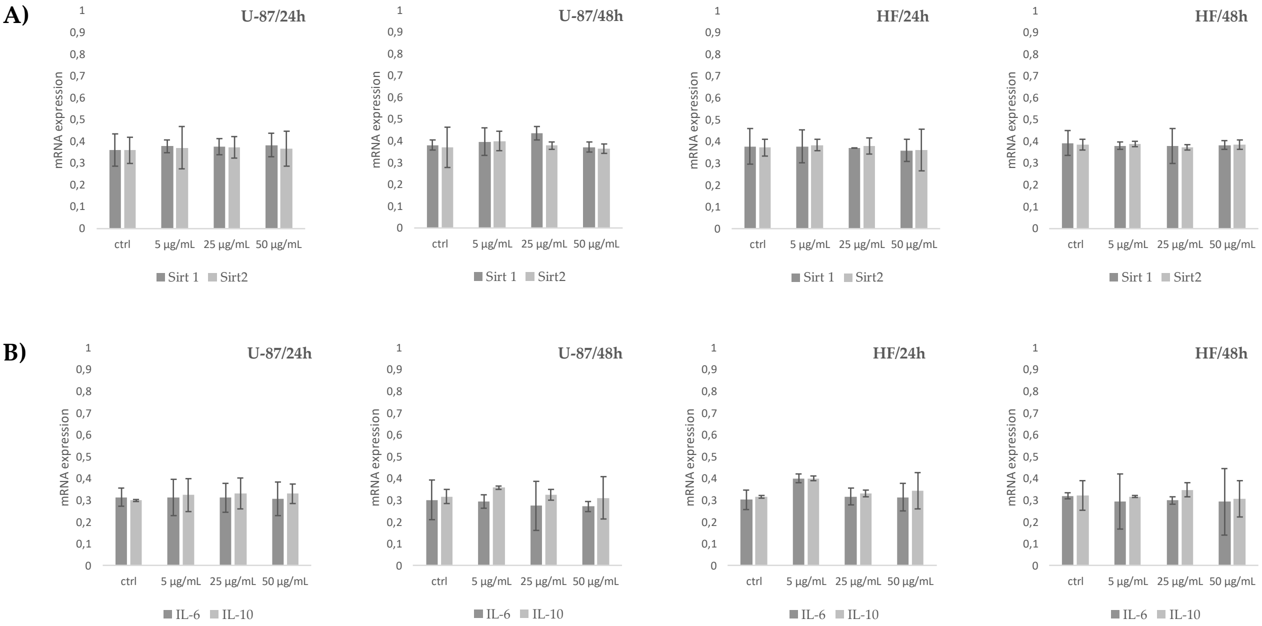

Figure 22. A) mRNA expression of Sirt1 and Sirt2 by real-time; B) mRNA expression of IL6 and IL10 by real-time in U-87 and human fibroblast (HF) cells after 24 and 48 h exposure times.
